# Revisiting ESKAPE Pathogens: virulence, resistance, and combating strategies focusing on quorum sensing

**DOI:** 10.3389/fcimb.2023.1159798

**Published:** 2023-06-29

**Authors:** Parvathy Venkateswaran, Sahana Vasudevan, Helma David, Adityan Shaktivel, Karthik Shanmugam, Prasanna Neelakantan, Adline Princy Solomon

**Affiliations:** ^1^ Quorum Sensing Laboratory, Centre for Research in Infectious Diseases (CRID), School of Chemical and Biotechnology, SASTRA Deemed to be University, Thanjavur, India; ^2^ Division of Restorative Dental Sciences, Faculty of Dentistry, The University of Hong Kong, Hong Kong, Hong Kong SAR, China

**Keywords:** ESKAPE, virulence, antimicrobial resistance, biofilm, quorum sensing

## Abstract

The human–bacterial association is long-known and well-established in terms of both augmentations of human health and attenuation. However, the growing incidents of nosocomial infections caused by the ESKAPE pathogens (*Enterococcus faecium*, *Staphylococcus aureus*, *Klebsiella pneumoniae*, *Acinetobacter baumannii*, *Pseudomonas aeruginosa*, and *Enterobacter* sp.) call for a much deeper understanding of these organisms. Adopting a holistic approach that includes the science of infection and the recent advancements in preventing and treating infections is imperative in designing novel intervention strategies against ESKAPE pathogens. In this regard, this review captures the ingenious strategies commissioned by these master players, which are teamed up against the defenses of the human team, that are equally, if not more, versatile and potent through an analogy. We have taken a basketball match as our analogy, dividing the human and bacterial species into two teams playing with the ball of health. Through this analogy, we make the concept of infectious biology more accessible.

## Introduction

**Figure 1:**
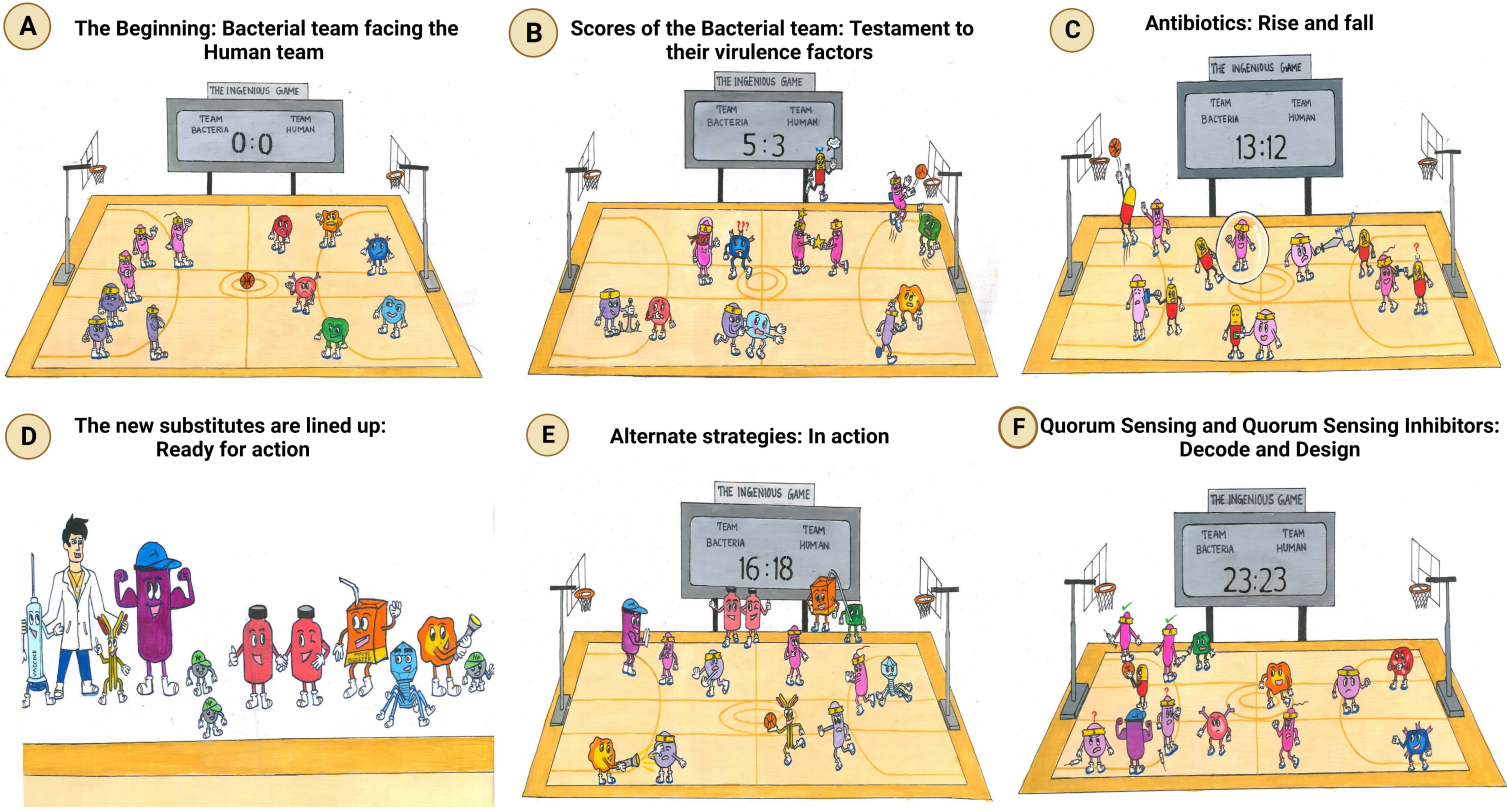
The ingenious game between team bacteria (ESKAPE) and team human. **(A)** The beginning: bacterial team facing the human team: bacterial team includes the Gram-positive *Enterococcus* sp., *Staphylococcus aureus*, and *Enterobacter* sp. and the Gram-negative *Klebsiella pneumoniae*, *Acinetobacter baumannii*, and *Pseudomonas aeruginosa*; the human team comprises macrophages, T-lymphocytes, B-lymphocytes, monocytes, eosinophils, and neutrophils. **(B)** Scores of the bacterial team: testament to their virulence factors. The bacterial players are rooted to the ground, closely adhering to the human body. The immune cells, however, cannot recognize them due to the masking effect of the bacterial capsule. To make things worse, another bacterium is spotted sharing its “special attribute” with their teammate. Ultimately, the bacterial team scores the goal, despite the efforts taken by the immune cell to block it. It is at this point that an antibiotic is spotted exclaiming its helplessness, being not recruited into the team. **(C)** Antibiotics: rise and fall. Although the antibiotics have achieved their goal, the bacteria have reduced their permeability, preventing the antibiotics from acting on them further. The bacterial players are also seen switching off the antibiotics by modifying them. Another bacterium is spotted in the act of slashing the functional antibiotic, rendering it inactive. Moreover, the antibiotic can no longer bind specifically to its target, as the bacterium has decoded the relentlessly used strategy of the human team and has modified the target. **(D)** The new substitutes are lined up: ready for action. The external coach, the researcher, is seen with a vaccine and monoclonal antibody on either side. Then comes the strong player representing various inhibitors—beta-lactamase inhibitor, efflux pump inhibitor, and conjugation inhibitor. Combinatorial drug molecules stand next to the highly versatile nanoparticles, winking and confirming their action plan. Next in the row is an immune booster. Adjacent to it, we see the grim-faced bacteriophage, which is waiting to take its toll! Lastly, we have the representative of antimicrobial light therapy holding a torch. **(E)** Alternate strategies: in action. The inhibitor is found to defend the antibiotic efficiently from the bacteria. Antimicrobial light therapy is affecting the bacteria. One bacterial player is alarmed at the entry of the combinatorial substitutes. Another bacterium is puzzled at the look of an immune cell drinking its energy potion! The monoclonal antibody has successfully recovered the ball of health from the bacterial team. Bacteriophage is doing its part by preventing bacterial players from entering human premises. **(F)** Quorum sensing and quorum-sensing inhibitors: decode and design. The bacterial players are spotted forming a protective shell (technically, biofilm) right below their goal post to defend their team. Among the four, two are caught communicating with each other, while the other pair is not, owing to the presence of a quorum-sensing inhibitor blocking their communication. On a closer look, the bacteria that cannot communicate with each other are equally unable to work with their injection (technically, express their virulence factor). This, in turn, has made them vulnerable to attack by the immune cell of the human team. Taking advantage of the current situation, the antibiotic has sneaked in and aims for the goal! Other players of the human team are seen guarding their goalpost against the entry of any bacterial player.

The incidence of bacterial players on the grounds of the human body is well-known (Ursell et al., 2012). The bacterial pathobionts play a significant role in assisting the human team in making them healthy by influencing stress levels, immune response, and cognition (Mohajeri et al., 2018). However, the opportunistic bacterial squad taking advantage of the immunocompromised state and the underlying dysbiosis in the human team are teamed up against the very human team, which they are an integral part of (Proença et al., 2017) (**Figure 1**). Studies show that antimicrobial resistance (AMR) causes more than 35,000 deaths annually and over 2.8 million recorded cases in the United States alone per year (Biggest Threats and Data | Antibiotic/Antimicrobial Resistance | CDC (Centers for Disease Control and Prevention); Martínez, 2014). Adding a feather to their cap, six prime players, namely, *Enterococcus* sp., *Staphylococcus aureus*, *Klebsiella pneumoniae*, *Acinetobacter baumannii*, *Pseudomonas aeruginosa*, and *Enterobacter* sp. (ESKAPE in short), have been shortlisted by the World Health Organization (WHO) owing to their mastery in the art of “escapism” (WHO, 2017). The human team is no less than the bacterial team, given its ability to defend itself by targeting diverse opponents consistently (Centers for Disease Control, 2019; Thakur et al., 2019). However, the bacterial team is versatile, wherein one species is reported to target multiple organs, just like an all-rounder including the lungs, kidneys, and skin (Bachman et al., 2011; Thomer et al., 2016; Okojie and Omorokpe, 2018). The highly coordinated human team is found to be constantly involved in keeping a check over any advances made by the bacterial team (Nicholson, 2016). Hence, this review aims to reinforce the human team by briefing about the strengths and strategies employed by the bacterial team and therefore augmenting the process of developing new strategies in preventing the bacterial team from scoring goals by infecting humans. It also attempts to capture this ingenious game between the bacterial team and the human team by recapitulating the various game plans and the substitutes employed by each team.

## ESKAPE: players’ biology and characteristics

### >Enterococcus sp.

*Enterococcus* sp. includes *Enterococcus faecium* and *Enterococcus faecalis*, ubiquitous pathogens with clinical relevance. They are Gram-positive and facultative anaerobes (Pendleton et al., 2013). As commensals, they are commonly found in the gut and modulate the immune system. They are opportunistic pathogens and translocate to different locations when there is an overgrowth in the gut due to antibiotic resistance or host inflammation (Krawczyk et al., 2021). Enterococci are associated with hospital-acquired infections, including catheter-associated urinary tract infections (CAUTIs), surgical site infections (SSIs), and bloodstream infections. Vancomycin-resistant enterococci (VREs) emerged in the 1980s and are still prevalent and estimated to cause 5,400 deaths in 2017 alone (Centers for Disease Control). Vancomycin-resistant *E. faecium* is on the WHO’s high-priority pathogen list (CDC, WHO).

### Staphylococcus aureus

*S. aureus* is Gram-positive and is considered one of the major pathogens. *S. aureus* is a skin commensal and becomes a pathogen in susceptible patients (Guo et al., 2020). *S. aureus* is found in wound infections and can cause multiple infections from soft tissue infections to infective carditis to bacteremia to fatal pneumonia (Tong et al., 2015). Methicillin-resistant *S. aureus* (MRSA) was isolated in 1961 and evolved with only 2 years of treatment. The spread of MRSA infection is so alarming that the number of deaths by MRSA has surpassed deaths by acquired immune deficiency syndrome (AIDS) and Parkinson’s disease, as per the report in 2012 (Lessa et al., 2012). The prevalence of MRSA is alarmingly even today and is clinically relevant. MRSA is also on the WHO’s high-priority pathogen list (CDC, WHO).

### Klebsiella pneumoniae

*K. pneumoniae* is a Gram-negative pathogen and belongs to the Enterobacteriaceae family. *K. pneumoniae* is most commonly associated with community-acquired pneumonia (Podschun and Ullmann, 1998; Piperaki et al., 2017). They are prominent extended-spectrum β-lactamase (ESBL) producers, making them a pathological threat in hospital settings. *K. pneumoniae* can infect multiple sites, including the lungs, urinary tract, blood stream, and brain. They are non-motile and encapsulated but present in both environments and on the surface of mammals. Hypervirulent strains of *K. pneumoniae* have also emerged (Russo and Marr, 2019), and carbapenem-resistant *K. pneumoniae* pose a significant threat. *K. pneumoniae* are intrinsically resistant to multiple antibiotics and found to cause sporadic cases worldwide (Lin et al., 2006).

### Acinetobacter baumannii

Carbapenem-resistant *A. baumannii* is one of the WHO critical priority pathogens that need immediate action. *A. baumannii* is a Gram-negative, opportunistic pathogen that can adapt to various hostile conditions. It can survive in dry conditions, erratic temperatures, and pH ranges, making it stay in the dynamic host and environmental conditions. *A. baumannii* is intrinsically resistant to antibiotics and also possesses resistant islands to impart resistance not only to antibiotics but also to metals and ammonium-based disinfectants. It can easily acquire β-lactamases, and most OXA carbapenemases are isolated in different clinical isolates of *A. baumannii*. It infects critically ill patients who are severely immunocompromised. It can cause hospital-acquired respiratory infections and urinary tract infections and is also present in wound infections. Considering its versatility and adaptability, *A. baumannii* is a tough nut to crack.

### Pseudomonas aeruginosa

Carbapenem-resistant *P. aeruginosa* is also one of the critical pathogens as defined by the WHO. *P. aeruginosa* is a Gram-negative, facultative anaerobe that infects immunocompromised patients and is often isolated from cystic fibrosis (CF) patients and burn patients (Moradali et al., 2017). *P. aeruginosa* can survive in harsh conditions and resist various antibiotics, mostly prominently fluoroquinolones (Livermore, 2002). It can cause infections at multiple sites, including the eye, skin, lungs, and urinary tract. Cystic fibrosis patients are most susceptible to *P. aeruginosa* infections from childhood (Malhotra et al., 2019), which is the prominent reason for mortality in CF adult patients (Doring et al., 2000). Nosocomial infections—ventilator-associated pneumonia, urinary tract infections, central line bloodstream infections, and surgical infections—are caused by *P. aeruginosa* and are considered the highest burden in healthcare settings (Lambert et al., 2011). Resistance to multiple classes of antibiotics combined with wide virulence factors to survive hostile conditions makes *P. aeruginosa* a mighty player to defeat.

### Enterobacter sp.

*Enterobacter* sp. is a group of Gram-negative pathogens, usually rod-shaped and facultative anaerobes. Like other pathogens, it is also often found in bacteremia, urinary tract infections, surgical site infections, and device-related infections (Davin-Regli and Pagès, 2015). *Enterobacter* sp. usually cannot be distinguished since it causes similar infections to other Gram-negative rod bacteria. However, ESBL-producing, carbapenem-resistant *Enterobacter* sp. is also one of the three critical pathogens listed by the WHO. *Enterobacter cloacae*, *Enterobacter asburiae*, and *Enterobacter hormaechei* are some of the clinically relevant species that have caused nosocomial outbreaks [(7) Clinical and pathogenesis overview of *Enterobacter* infections | Request PDF].

The commonality between the bacterial team players is their prominence in multidrug resistance, targeting immunocompromised patients causing nosocomial outbreaks, ability to adapt and survive in harsh environments, and translocating from one site to another. Understanding the virulence mechanism and resistance pathways is the need of the hour to devise strategies to tackle them effectively.

## Virulence factors: strengths of the bacterial team

**Figure 2:**
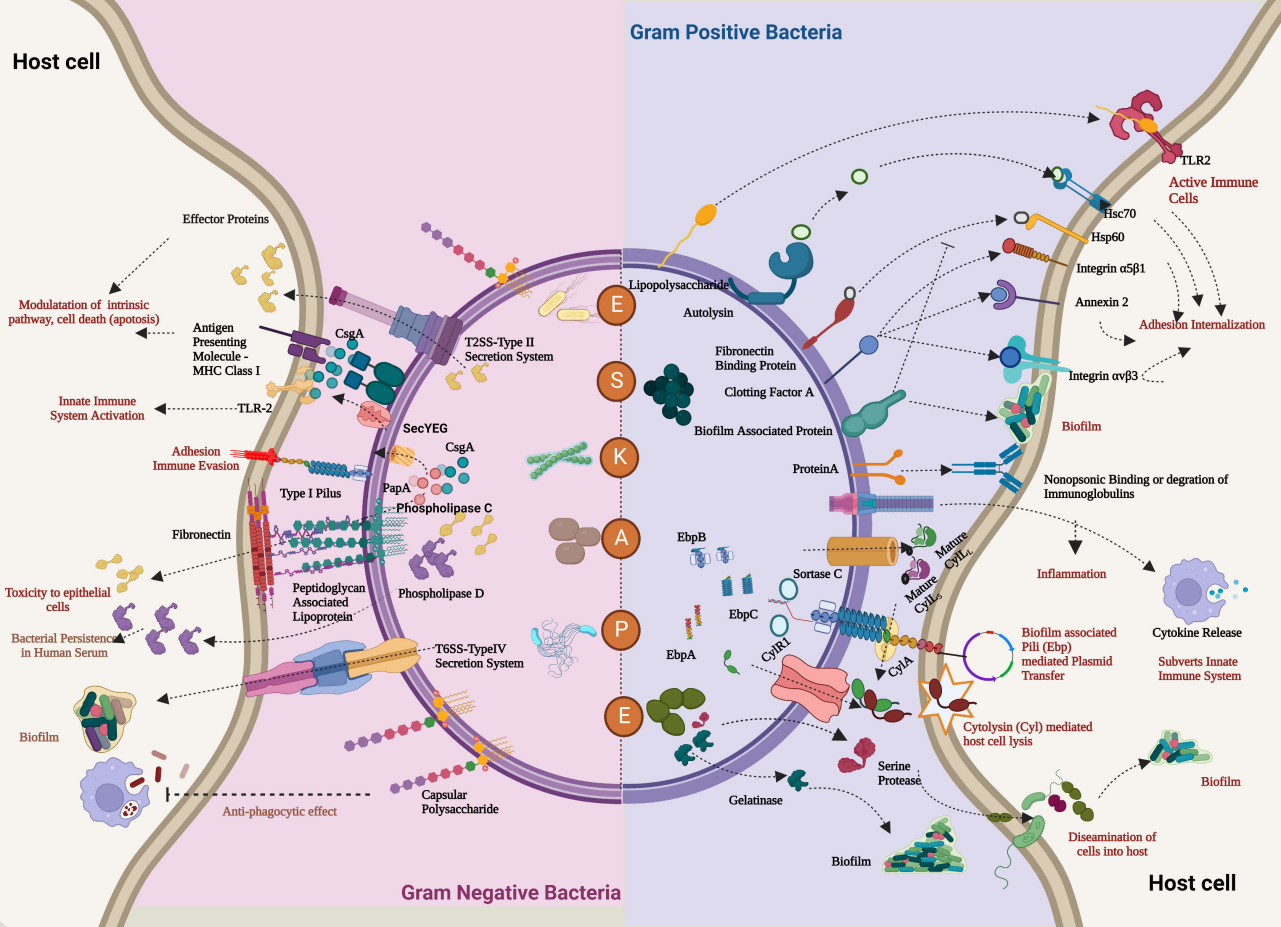
Comprehensive overview of the virulence factors of the ESKAPE pathogens. In the case of both Gram-positive bacteria (*Enterococcus faecalis* and *Staphylococcus aureus*) and Gram-negative bacteria (*Klebsiella pneumoniae*, *Acinetobacter baumannii*, *Pseudomonas aeruginosa*, and *Enterobacter* sp.), the host evasion is orchestrated by the recurring events: adhesion to the host cells, Degradation by a range of degradative enzymes and toxins establishes biofilm to trigger the innate immune pathways and further deteriorates the cellular homeostasis of the host cell. In addition, these bacteria also transfer their virulence factors through horizontal gene transfer, which leads to persistent infections. Created with BioRender.com.

The bacterial team has attained ascendancy in the game through a detailed pathogenesis process. The pathogenesis process is a multilevel and complex process involving various factors to establish a successful infection of the host (Wilson et al., 2002). Even though the elements and approach of pathogens vary, a similar pattern is followed. To mark their territory in the host, the bacterial members team up through a strong adhesion between them and the host team (Ribet and Cossart, 2015). Thus, the first step is the adhesion of the bacteria among themselves: auto-aggregation, microcolony formation, and ultimately biofilm formation, followed by solid adhesion to the host through mucosal surfaces. The adhesion step is crucial for bringing dysbiosis to the host microbiota and colonizing and invading the host cells (Pizarro-Cerdá and Cossart, 2006). Once they have adhered, the bacterial cells invade the host cells and release different toxins—proteins, enzymes, and siderophores—to affect the healthy host cells and evade the immune system (Siegel and Weiser, 2015). **Table 1** elaborates the reported key genes involved in every step of the virulence process of ESKAPE pathogens. A part of the invaded bacteria goes to a quiescent state, termed “persisters”, to invoke recalcitrant infections later (Vasudevan et al., 2022). Understanding the underlying mechanisms dictating such survival mechanisms has been of utmost importance in recent days (Kaushik et al., 2022). Pathogens use the host environmental factors to drive this process and resist antibiotics (Hakansson et al., 2018). Several pathways and dedicated regulatory networks are involved in the pathogenesis (de Macedo et al., 2021). **Figure 2** captures the virulence factors of each of the ESKAPE pathogens briefed below.

**Table 1:** Summary of the known virulence factors of ESKAPE organisms.

Process involved with virulence	Bacterial species	Associated molecule(s)	Role in pathogenesis	Gene(s)	Reference(s)
Adhesion	*Enterococcus* sp.	Enterococcal surface protein (ESP)	Enhances persistence in UTI	*esp*	(Toledo-Arana et al., 2001)
		Aggregation substance	Facilitates donor–recipient contact during conjugation	*asa1*, *asp1*, and *acs10*	(Rozdzinski et al., 2001; Sava et al., 2010)
		MSCRAMMAce	Binds to collagen	*ace*, *acm*, and *scm*	(Hendrickx et al., 2009)
		Capsule	Adheres to ECM	*cpsF*, *cpsC*, *cpsD*, *cpsE cpsG*, and *cpsI*	(Thurlow et al., 2009)
	*Staphylococcus aureus*	MSCRAMMs	Protein that binds to collagen	*ena*, *cna*, *ebps*, and *bbp*	(Firoozeh et al., 2020)
		Fibronectin binding proteins A, B	Aids cell adhesion	*fnbA*, *B*	(Firoozeh et al., 2020)
		Clumping factors, A and B	Facilitates the colonization of protein-coated biomaterials	*clfA*, *B*	(Firoozeh et al., 2020)
	*Klebsiella pneumoniae*	Type I and III fimbriae	Facilitates adhesion	*fimA*, *fimH*, *mrkA*, and *mrkD*	(Alcántar-Curiel et al., 2013)
		Type VI protein secretion system	Aids cell invasion and *in vivo* colonization	*icmF1* and *icmF2*	(Hsieh et al., 2019)
	*Acinetobacter baumannii*	Capsule	Facilitates cell–cell adhesion	*pglC* and *ptk*	(Murray et al., 2017)
	*Pseudomonas aeruginosa*	Type IV pili (TFP)	Facilitates adhesion	*pilU*	(Whitchurch and Mattick, 1994; Choy et al., 2008)
		Alginate	Enhances adhesion to solid surfaces	*algC*, *algD*, and *algT*	(Muhammadi and Ahmed, 2007)
	*Enterobacter* sp.	Type VI secretion system	Aids cell adherence and facilitates colonization	*clpV1* and *clpV2*	(Soria-Bustos et al., 2020)
		Enterobactin	Improves adsorption to metal surfaces	*entB*	(Upritchard et al., 2011)
Ability to produce enzymes and toxins	*Enterococcus* sp.	Hemolysin	Cytolytic protein that cleaves the erythrocytes	EF_0700	(Zhang et al., 2007)
		Gelatinase	Cleaves gelatin, collagen, casein, hemoglobin, and other peptides	*gelE*	(Maasjost et al., 2019)
		Hyaluronidase	Cleaves hyaluronate	*hyl_Efm_*	(Maasjost et al., 2019)
		Cytolysin	A two-peptide bacteriocin that forms pores	*cylL_L_*, *cylL*, *cylM*, *cylB*, and *cylA*	(Maasjost et al., 2019)
	*S. aureus*	Hemolysins α, β, γ, and δ	Cleaves erythrocytes	*hla*, *hlb*, *hld*, and *hlg*	(Wang et al., 2014; Motamedi et al., 2018)
		Hyaluronidase	Enhanced intracellular survival and inhibition of pro inflammatory cytokine expression	*HysA*	(Ibberson et al., 2014)
		Collagenase	Cleaves collagen	yhbU_2	(yhbU_2–collagenase-like protease–*S. aureus*–yhbU_2 gene and protein)
		Panton-Valentine Leukocidin	Forms pores	*lukS-PV* and *lukF-PV*	(Melles et al., 2006)
		Staphylokinase	Activates host plasminogen	*sak*	(Sako and Tsuchida, 1983)
	*K. pneumoniae*	Hemolysin	Cleaves erythrocytes	*hly*	(Pereira and Vanetti, 2015; Esmaeel and Sadeq, 2018)
		Phospholipase D	Cleaves phospholipids	*pld1*	(Lery et al., 2014)
	*A. baumannii*	Phospholipase (PLC and PLD)	Cleaves phospholipids	*pld*	(Lee et al., 2017; Murray et al., 2017)
		CipA	Binds to host plasminogen and can improve penetration into endothelial monolayers	*cipA*	(Koenigs et al., 2016)
	*P. aeruginosa*	Enterotoxin	Forms pores in the cell membrane	*tox* A	(Pollack, 1984; Dapgh et al., 2019)
		Phospholipase	Cleaves phospholipids	*pcl*H	(Dapgh et al., 2019)
	*Enterobacter* sp.	Hemolysin	Cleaves erythrocytes	*αhly*	(Burgos, 2010)
		PrtA, B, and C family protease	Cleaves proteins	*prtA*, *prtB*, and *prtC*	(Ghigo and Wandersman, 1992)
Ability to evade the immune system	*Enterococcus* sp.	Capsule	Provides a barrier	*cpsF*, *cpsC*, *cpsD*, *cpsE cpsG*, and *cpsI*	(Thurlow et al., 2009)
	*S. aureus*	Type 1 capsular polysaccharide	Provides a barrier	*cap1*	(Luong et al., 2002)
		Clumping factor	Inhibits phagocytic engulfment	*clfA*, *B*	(Higgins et al., 2006)
		Teichoic acid	Aids in disguise	*tarB*, *tarD*, *tarF*, *tarIJ*, and *tarH*	(D’Elia et al., 2006)
	*K. pneumoniae*	Capsular polysaccharide-mediated factors	Provides a barrier	*Cps*	(Hsu et al., 2016)
	*A. baumannii*	Lipopolysaccharide (LPS)	Binds to the CD14/TLR4/MD2 receptor complex of immune cells	*lpxA*, *lpxC*, and *lpxD*	(Moffatt et al., 2013; Lee et al., 2017)
		Outer membrane protein A (OmpA), Omp33-36, and Omp22	Modulates autophagy	*OmpA*, *Omp33-36*, and *Omp 22*	(Rumbo et al., 2014)
	*P. aeruginosa*	Alkaline protease	Cleaves proteins	*aprA*	(Iiyama et al., 2017)
		Elastase	Cleaves elastin	*lasB*	(Rust et al., 1996)
	*Enterobacter* sp.	Peptidoglycan	Regulates the accessibility of pathogen-associated molecular patterns (PAMPs)	–	–
Horizontal gene transfer	*Enterococcus* sp.	Pili	Facilitates conjugation	*ebp*, *pila*, and *pilb*	(Hendrickx et al., 2009)
	*S. aureus*	Sortase A	Links surface proteins to peptidoglycan	*Sau*‐SrtA	(Khare and Narayana, 2017)
	*K. pneumoniae*	Pilin	Facilitates conjugation	*ecpA*, *ecpR*, and *ecpB*	(Alcántar-Curiel et al., 2013)
	*A. baumannii*	Type VI protein secretion system	Facilitates conjugation	*tss* and *tag*	–
	*P. aeruginosa*	Type IV pili	Facilitates conjugation	*pilU*	(Whitchurch and Mattick, 1994)
	*Enterobacter* sp.	–	–	*-*	–
Iron acquisition system	*Enterococcus* sp.	Fur	Regulates the uptake of free iron and maintains iron homeostasis	*Fur*	(Latorre et al., 2018)
	*S. aureus*	Siderophores, staphyloferrin A (SA), and staphyloferrin B (SB)	Acquires free iron	*sbn sfa*	–
		Hts, Sir transporters	Staphyloferrin uptake	*sirA hts*	(Beasley et al., 2011)
	*K. pneumoniae*	Siderophores, aerobactin (*iuc*), and salmochelin (*iro*)	Acquires free iron	*iuc* and *iro*	–
	*A. baumannii*	Acinetobactin NfuA	Acquires free iron	–	–
	*P. aeruginosa*	Siderophores pyoverdines, PVDI, PVDII, and PVDIII; FpvAI and FpvB	Acquires free iron Outer membrane transporters	*fpv*	(Hartney et al., 2013; Bonneau et al., 2020)
	*Enterobacter* sp.	Yersiniabactin	Acquires free iron	*irp1*, *irp2*, and *fyuA*	–
Ability to form a biofilm	*Enterococcus* sp.	Capsule	Mediates cell–cell adhesion	*cpsF*, *cpsC*, *cpsD*, *cpsE cpsG*, and *cpsI*	(Thurlow et al., 2009)
		Cell wall polysaccharide	Contributes to form the extracellular matrix	*epa*	(Hancock et al., 2014)
		Aggregation substance	Increases surface hydrophobicity	*asa1*, *asp1*, and *acs10*	(Rozdzinski et al., 2001; Sava et al., 2010)
	*S. aureus*	Aggregation substance	Increases surface hydrophobicity	*Asa1*	(Sussmuth et al., 2000)
	*K. pneumoniae*	Capsular polysaccharide	Mediates cell–cell adhesion	–	–
		Type III fimbriae	Maintains contact by promoting fimbrial adhesion	*MrkA*, *MrkBC*, *MrkD*, and *MrkF*	(Schroll et al., 2010; Johnson et al., 2011)
	*A. baumannii*	Capsular polysaccharide	Helps in capsule polymerization and assembly which aids in biofilm formation	*Ptk* and *epsA*	(Russo et al., 2010)
	*P. aeruginosa*	Type IV pili	Maintains contact	*pilU*	(Whitchurch and Mattick, 1994)
	*Enterobacter* sp.	Capsule	Mediates cell–cell adhesion	–	–

"-" denotes insufficient information.

### Biofilm formation

Biofilms are commonly associated with increasing antibiotic resistance due to their ability to protect pathogens from antibiotics and other environmental stress factors. Biofilms act as a physical barrier that prevents the diffusion of antimicrobials and upregulates specific biofilm-associated virulent genes contributing to antimicrobial resistance (Tuson and Weibel, 2013; Bowler et al., 2020). Understanding the course of biofilm formation and its regulation could be instrumental in preventing biofilm formation, re-structuring, and disintegrating existent biofilms (Dale et al., 2017). Biofilm formation predominantly involves four stages/moves by the bacterial team: 1) adhesion, 2) microcolony formation, 3) biofilm growth and maturation, and 4) dispersal.

#### Move 1: adhesion

The first and foremost step in forming a robust biofilm is surface adhesion. For instance, targeting this phase of biofilm formation, which depends on various factors, including surface charge, roughness, wettability, stiffness, topography, and bacterial motility, through different physical and chemical methods has been proven to be successful (Solanki et al., 2018; Zheng et al., 2021; Uneputty et al., 2022). Various adhesion-related genes, including the ones coding for aggregation substance *agg1*, collagen binding proteins *ace*, and enterococcal surface protein *esp*, were highly prevalent and were found to play a significant role in determining the virulence of *E. faecalis* clinically (Strateva et al., 2016). The deletion of *ebp*—the pilus-encoding gene—is reported to significantly impact the virulence and biofilm-forming ability of *E. faecalis* (Sillanpää et al., 2010). Another study by Soares et al. identified that genes that aid adhesion—*esp* and *agg*—are crucial for augmenting biofilm formation in the clinical isolates of *Enterococcus* sp. (Soares et al., 2014). However, a former study has observed enterococcal biofilms without esp, highlighting that this factor is not essentially indispensable for biofilm formation (Kristich et al., 2004). For a more detailed overview of enterococcal biofilm formation, the readers are directed to the review by Ch’ng et al. (Ch’ng et al., 2018). In the case of *S. aureus*, genes that encode fibronectin-binding protein (*fib*, *fnbA*, and *B*), clumping factors (*clfA* and *B*), elastin-binding protein (*ebp*), and serine-aspartate repeat family (*sdr*) are known to mediate surface adhesion (Chen Q et al., 2020). A recent study identified the presence of *clfB*, *ebp*, and *sdrD* in multidrug-resistant *S. aureus* strains isolated from periodontal lesions of patients and found an increased incidence of biofilms among these isolates (Uribe-García et al., 2021). Along these lines, it was reported that reduced expression of adhesion-related genes *agr* and *sdr* further diminished the ability of MRSA to form biofilms (Iwata et al., 2021).

Similarly, genes that promote adhesion, including the ones that encode type III fimbriae *fim*, a homolog of enterococcal *ebp* and protein secretion system *icm*, have proven to be attractive targets to reduce the biofilm formation ability of the opportunistic bacteria *K. pneumoniae* (Schroll et al., 2010; Alcántar-Curiel et al., 2013; Vuotto et al., 2014). Reiterating surface adhesion’s crucial role in biofilms’ structural organization, Raffaella Campana et al. proved that reduced bacterial adhesion impaired the biofilm-forming ability of *K. pneumoniae* in medical devices (Campana et al., 2017). Nevertheless, another study identified a direct correlation between the strength of adhesion and the biofilm-forming ability of *K. pneumoniae*, supporting the idea of targeting the first step in biofilm formation for attenuating virulence (Lenchenko et al., 2020). Taken together, it can be concluded that adhesion determines the strength of biofilms, and therefore, targeting this could prove to be a promising strategy for tackling ESKAPE-mediated infections. However, many factors influencing adhesion, including the surface that bacteria adhere upon, multi-species environment, and types of appendages employed for adhesion, should be considered while deciding upon the targets and designing novel strategies against these pathogens.

#### Move 2: microcolony formation

The bacterial cells adhered to the surface and then proliferate and form structurally organized micro-colonies embedded in a matrix of polysaccharides, proteins, lipids, and nucleic acids (Karygianni et al., 2020). The extracellular polysaccharides influence the architecture and the immediate surroundings of the bacterial cells by affecting the hydrophobicity, mechanical stability, charge, porosity, water content, and other essential nutrients. Interestingly, oxygen, hydrogen, and nutrient gradients also form during this stage, creating different microenvironmental conditions within the biofilm (Petrova et al., 2012). This phase, in which solitary bacterial cells come together to form a microcolony, is crucial in understanding biofilm formation and targeting novel preventative and therapeutic strategies. Recent studies identified the ability of *Enterococcus faecalis* to develop distinct microcolonies on the entire valvular regions. However, these colonies’ potential to advance and cause infection is still less explored (Barnes et al., 2022). In the case of *S. aureus*, the matrix is predominantly proteinaceous due to Bap protein. Bap protein has been identified to be a crucial player in promoting biofilm formation in *S. aureus* (Taglialegna et al., 2016). In addition, various other proteins, including FnBPA, FnBPB, and SdrC, have been shown to contribute to microcolony formation (Schilcher and Horswill, 2020). *mifR* is one of the significant factors contributing to microcolony formation in *P. aeruginosa*. Petrova et al. identified the importance of pyruvate and its utilization through fermentation to promote the development of microcolonies (Petrova et al., 2012). Although the specific genes and regulatory mechanisms dictating microcolony formation of ESKAPE pathogens are not fully understood, the evidence points to the importance of understanding and manipulating the same to better fight against these pathogens. Considering that this step is crucial in determining the structural organization of the biofilms, tampering with this phase could also help bring down the tower-like and mushroom-shaped biofilms (Dale et al., 2017).

#### Move 3: biofilm maturation and dispersal

Biofilm maturation is triggered by the accumulation of extracellular polymeric substances (EPS), eDNA, formation of channels for waste disposal and nutrient exchange, varying ionic concentrations, and most, importantly, quorum-sensing signals (Moormeier and Bayles, 2017; Wang T. et al., 2019). It has also been reported that it is at this phase that the genes responsible for flagellar development are downregulated, satisfying the need for building a stable biofilm architecture (de Kievit, 2011). To start with the case of *E. faecalis*, the crucial role played by eDNA in biofilm maturation has been re-iterated continuously. It has been reported that the reduction in eDNA levels, by either cleaving the eDNA by Dnase or by preventing its release by inhibiting AtlA, significantly disrupts the enterococcal biofilm and makes it susceptible to treatment (Yu et al., 2019). Staphylococcal biofilms, however, are identified to exist in two different microcolony structures based on the expression of *cidABC* and *irgAB* (Moormeier and Bayles, 2017). Various EPS components, including Psl, Pel, alginate, eDNA, and the proteinaceous components, have been reported to play specific roles in forming and maturing *Pseudomonas* biofilms (Wei and Ma, 2013). Overall, infectious biofilms often observed in clinical settings have been known to be highly matured, and targeting such structurally robust biofilms has been a difficult challenge. Various modern advancements in the field of therapeutics—CRISPR technology, quorum-sensing inhibition, and antimicrobial peptides, among others—have proven to be promising despite the need for extensive research in the respective domains (Jiang et al., 2020; Nadar et al., 2022). Inducing the dispersal of individual bacterial cells embedded in the EPS has also been instrumental in tackling the infection, considering the increased susceptibility of planktonic cells to antibiotics and other antimicrobial strategies. This strategy, however, also has an inherent risk of speeding up bacterial colonization by actively triggering biofilm dispersal. A deeper understanding of the dispersal mechanisms of the ESKAPE pathogens would be beneficial in translating various strategies to the bedside.

### Colonization and invasion

The whole point of adhering to the host team is to infiltrate the human team and render them insufficient (Pizarro-Cerdá and Cossart, 2006). The pathogens must overcome the ever-dynamic physiological host environment—temperature, pH, and presence of other components—to colonize successfully. ESKAPE pathogens are mostly commensal-turned or hospital-acquired pathogens that affect the gut and cause bacteremia, oral infections, wound infections, and urinary tract infections. As can be seen, each host niche is unique, and to establish infection, host barriers are to be surpassed. The most prominent barrier is the acidic pH (2 to 5). *Enterococcus* sp. has adapted to tolerate acidic pH (Başaran et al., 1998). Also, commensals are reduced due to the antibiotic’s treatment, leaving the way for *Enterococcus* sp. to flourish. Adherence to the host site strongly supports the translocation to other sites, including blood, lymph nodes, blood, and spleen (Fiore et al., 2019). A similar trajectory is followed by *S. aureus*, where it has to overcome the host barriers to colonize the host (Liu, 2009). The breach of the intact microbiota, immune system evasion, and immune cell colonization support successful colonization. Both *Enterococcus* sp. and *S. aureus*, belonging to the Gram-positive group, teichoic acids, have primarily played a role in the successful colonization of the host. Once the propagation in the host site begins, the pathogens start to produce virulence factors—especially toxins and enzymes to disarm the host immune system and bring damage to the host. Taking an aggressive stance by making extracellular enzymes and toxins damage the host tissue has been customary in easing this process (Upadhyaya et al., 2009; Newman et al., 2017). Hemolysin encoded by EF_0700 gene is a potent toxin that cleaves the erythrocytes found in *Enterococcus* sp. (Zhang et al., 2007). Similarly, gelatinase, encoded by *gelE*, cleaves the host gelatin, collagen, casein, hemoglobin, and peptides. *hyl_efm_*, which encodes hyaluronidase, cleaves hyaluronate present in the connective tissues (Maasjost et al., 2019). *Enterococcus* sp. produces cytolysins, which are two-peptide bacteriocins that form pores and damage the host tissue, encoded by gene cassettes *cylL_L_*, *cylL*, *cylM*, *cylB*, and *cylA* (10.2217/fmb-2021-0212). Hemolysins α, β, γ, and δ, which cleave erythrocytes encoded by *hla*, *hlb*, *hld*, and *hlg*, also present in *S. aureus* (Wang et al., 2014; Motamedi et al., 2018). *hysA* encodes hyaluronidase (Ibberson et al., 2014), *ybhu_2* encodes collagenase, and *lukS-PV* and *lukF-PV* code Panton-Valentine Leukocidin, which forms pores (Melles et al., 2006) in the host system aid for *S. aureus* colonization process. Staphylokinase encoded by *sak* binds with the host plasminogen resulting in the plasmin enzyme, which essentially aids in the *S. aureus* penetration into the tissues (Sako and Tsuchida, 1983). In the case of Gram-negative pathogens, phospholipase D production, which cleaves phospholipase and hemolysin, is commonly used to damage the host. *hly* and *pld1* genes in *K. pneumoniae* encode hemolysin (Pereira and Vanetti, 2015; Esmaeel and Sadeq, 2018) and phospholipase D (Lery et al., 2014), respectively. In *A. baumannii*, *pld* gene encodes phospholipases (PLC and PLD) (Lee et al., 2017; Murray et al., 2017), *cipA* gene encodes CipA, which has a similar function as staphylokinase, binds to plasminogen, and promotes penetration of *A. baumannii* in the endothelial monolayers (Koenigs et al., 2016). *toxA* encodes endotoxin in *P. aeruginosa*, which also forms pores in the cell membrane (Pollack, 1984; Dapgh et al., 2019) and also produces phospholipase encoded by *pclH* (Dapgh et al., 2019). In *Enterobacter* sp., hemolysin is encoded by *hly* (Burgos, 2010), whereas PtrA, B, and C families of proteases are encoded by *prtA*, *prtB*, and *prtC*, which cleave host proteins and promote colonization of the host (Ghigo and Wandersman, 1992).

Evading the immune system, the defending team is the next crucial step after getting hold of the ball (Finlay and McFadden, 2006). Different capsular serotypes, peptidoglycan, teichoic acid, and protein A have helped bacteria escape from the host humoral and cellular innate defenses by fooling them and turning them down (Leitão, 2020). Capsular polysaccharides have an evasion process to escape the immune system. These capsular polysaccharides surround the bacterial surface and evade complement activation, phagocytic killing, and opsonization (Merino and Tomás, 2010). *cpsF*, *cpsC*, *cpsD*, *cpsE*, *cpsG*, and *cpsI* in *Enterococcus* sp. encode the capsule (Thurlow et al., 2009). *cap1* in *S. aureus* encodes type 1 capsular polysaccharide (Luong et al., 2002), and *cps* in *K. pneumoniae* (Hsu et al., 2016) and cps gene clusters in *A. baumannii* encode the capsule polysaccharide (Singh et al., 2019). In addition, clumping factors and teichoic acids encoded by *clfA* and *B* (Higgins et al., 2006) and *tarB*, *tarD*, *tarF*, *tarIJ*, and *tarH* (D’Elia et al., 2006) inhibit phagocytic engulfment in *S. aureus*. Cell membrane components play an essential role in the immune evasion process. In *A. baumannii*, *lpxA*, *lpxC*, and *lpxD* encode lipopolysaccharide, which effectively binds to the CD14/TLR4/MD2 receptor complex of immune cells and subverts its action (Moffatt et al., 2013; Lee et al., 2017). Outer membrane proteins modulate autophagy, which is mediated by *ompA*, *omp33-36*, and *omp22* genes encoding for OmpA, Omp 33-36, and Omp-22, respectively. Alkaline protease encoded by *aprA* (Iiyama et al., 2017) and elastase encoded by *lasB* (Rust et al., 1996) evade the immune system by cleaving immunoglobulins, inactivating the complement system and several cytokines (TNF, IFN, IL1, and IL6).

Further, to improve the chances of winning, the bacterial team strengthens itself through horizontal gene transfer (Lerminiaux and Cameron, 2019). This trait has empowered the bacteria not primarily equipped with specific virulence factors and has posed an arduous challenge to the opponent team. For instance, a recent study reported the transfer of various virulence-related genes in *Staphylococcus* sp., which increased its pathogenicity (Smith and Andam, 2021). Bacterial cell wall appendages promote horizontal gene transfer to a large extent. Pili, hair-like appendages, primarily facilitate conjugation and transfer antibiotic resistance genes from one bacterium to another (Sun, 2018). *ebp*, *pila*, and *pilb* genes in *Enterococcus* sp. (Hendrickx et al., 2009); *ecpA*, *ecpR*, and *ecpB* in *K. pneumoniae* (Alcántar-Curiel et al., 2013); and *pilU* in *P. aeruginosa* (Whitchurch and Mattick, 1994) encode pili that facilitate conjugation. In addition, Sortase A enzyme of *S. aureus*, encoded by s*au*‐srtA that links the surface proteins to peptidoglycan (Khare and Narayana, 2017) and the type VI secretion system, also play a role in horizontal gene transfer.

The bacterial team also constantly competes with the human team for resources such as free iron (Kronstad and Caza, 2013). Iron is an essential metal that bacterial pathogens require for multiple processes like respiration, metabolism, and other iron-dependent cellular processes. The iron requirement is huge for bacteria, and iron acquisition is a prerequisite to sustaining them in the host environment. Similarly, iron is a co-factor for multiple enzymatic processes in the human system. They are also found in metalloprotein heme complexes: hemoglobin, myoglobin, catalases, cytochromes, and aconitase as Fe-S clusters. Immune cells, macrophages, and other cells are used as iron transporters during iron deficiency; thus, iron homeostasis is maintained. Hence, iron competition is fierce between the pathogens and the host. Bacteria have developed various mechanisms to sequester available iron from the environment. Ferric uptake regulatory proteins (Fur) are essential for maintaining iron homeostasis in most bacterial pathogens, especially *Enterococcus* sp. (Latorre et al., 2018). In *S. aureus*, sbn and sfa encode siderophores staphyloferrin A (SA) and staphyloferrin B (SB). *K. pneumoniae* has *iuc* and *iro* genes that encode siderophores aerobactin (*iuc*) and salmochelin (*iro*). Acinetobactin NfuA of *A. baumannii* and fpv in *P. aeruginosa* encode siderophores: pyoverdines (PVDI, PVDII, and PVDIII) and FpvAI and FpvB (Hartney et al., 2013; Bonneau et al., 2020). Yersiniabactins encoded by *irp1*, *irp2*, and *fyuA* are responsible for iron acquisition.

Also, the constantly evolving host–bacterial interactions determine the extent of the underlying pathogenesis by influencing the process of adherence, invasion, and biofilm formation. For instance, Scherr TD et al. identified the differential expression of genes associated with biofilm formation in *S. aureus* when exposed to different subsets of immune cells, aiding in its persistence (Scherr et al., 2013). In addition to the immune factors, the host microenvironment *in vivo* influences the biofilm’s nature. Rahman MUA et al. identified the role of free collagen in determining the viscoelasticity of *P. aeruginosa* biofilms. Understanding biofilms’ stability and homogeneity and the way the host environment dictates it could prove instrumental in replicating *in vivo* conditions more accurately and in targeting biofilms more efficiently (Rahman et al., 2021). A recent study reported the role of interaction between host fibronectin and peptidoglycan-associated protein of *A. baumannii* in biofilm formation. It explored the possibility of therapeutic targeting of this bacterial protein to augment the immune response (Solanki et al., 2023).

One other key strategy is to form biofilms by aggregating with each other within and across species. A plethora of evidence suggests biofilm formation aggravates the infection by improving cell adhesion, colonization, and horizontal gene transfer. Significant factors, including the capsule, aggregation substance, pili, and fimbriae, are reported to be associated with assisting biofilm formation. In particular, the capsule contributes toward shielding the bacteria from various harsh conditions, including pH, temperature, ultraviolet (UV) radiation, antibiotics, and poor nutrients, by acting as physical barriers and by providing a confluent microenvironment, thereby sustaining survival and metabolism (Yin et al., 2019; Vor et al., 2020). Various stress conditions, including pH, temperature, and oxygen availability, are crucial in triggering biofilm formation in certain bacteria, such as *S. aureus*, *P. aeruginosa*, and *Enterobacter* sp. (Hoštacká et al., 2010; Gupta et al., 2016; Chu et al., 2018). It is essential to emphasize that more than one virulence factor generally acts in synergy to introduce the infection (**Figure 1B**) successfully.

## Antibiotics: the substitutes

Time has arrived for the human team to employ innate and adaptive immune strategies to prove its competence against the bacterial squad, which has skillfully scored well in the first half of the match. Since relying only on the immune cells has proven inadequate, recruiting substitutes to strengthen the team has been hypothesized to be a good strategy (**Figure 1C**). Arsphenamine, a toxic dye, was one of the first substitutes that signed up for the match. Despite the effectiveness of this dye in treating syphilis, arsphenamine has not been employed widely owing to its toxicity to human cells, which ultimately kills the patients (FROM DYES TO PEPTIDES: THE EVOLUTION OF ANTIBIOTIC DRUGS | SCQ). Scrutinizing the target specificity and sensitivity of the drug is a crucial step in developing novel drug classes. Conscribing penicillin, the serendipitous drug, has manifested itself as one of the finest action plans until recently (Gaynes, 2017). Since then, an extensive range of antibiotics has been synthesized from various sources targeting Gram-positive and Gram-negative bacteria. Targeting the molecular mechanisms involved in cell growth (bacteriostatic) and bacterial survivability (bactericidal) has been authenticated to be an effective method (Pankey and Sabath, 2004). Antibiotics have proven to be a valuable addition to the human team by scoring goals (restoring “health”) and reducing the bacteria’s activity by binding with them.

Nonetheless, this effect was not persistent. The delimiting nature of monotherapy to tackle the infection has laid the foundation for recruiting more antibiotics against the skillful bacterial team. In this instance, the game started to change with a much-unexpected twist. Indiscriminate employment of players uninformed about the opponent team, such as the non-specific antibiotics, started turning down the strength of the human group (Om et al., 2016). To exacerbate the situation, the bacterial team has started unveiling their opponent’s strategies and devising new mechanisms to fool the combatants (Santajit and Indrawattana, 2016). Using the same class of antibiotics multiple times has been reported to be one major pitfall that alerted the bacterial team to decode our game plan. Nevertheless, modifying the scaffolds of the previously designed antibiotics has raised their potency and increased the chances of winning for the human team. Still, the bacterial team has formulated innovative plans, such as the utilization of efflux pumps and enzymes, chemical modification of drugs and the target, and alteration in membrane permeability, leading to the development of the pressing issue of antimicrobial resistance, the central feature that has raised the stature of the ESKAPE pathogens.

## Antibiotic resistance mechanisms: the masterstroke

**Figure 3:**
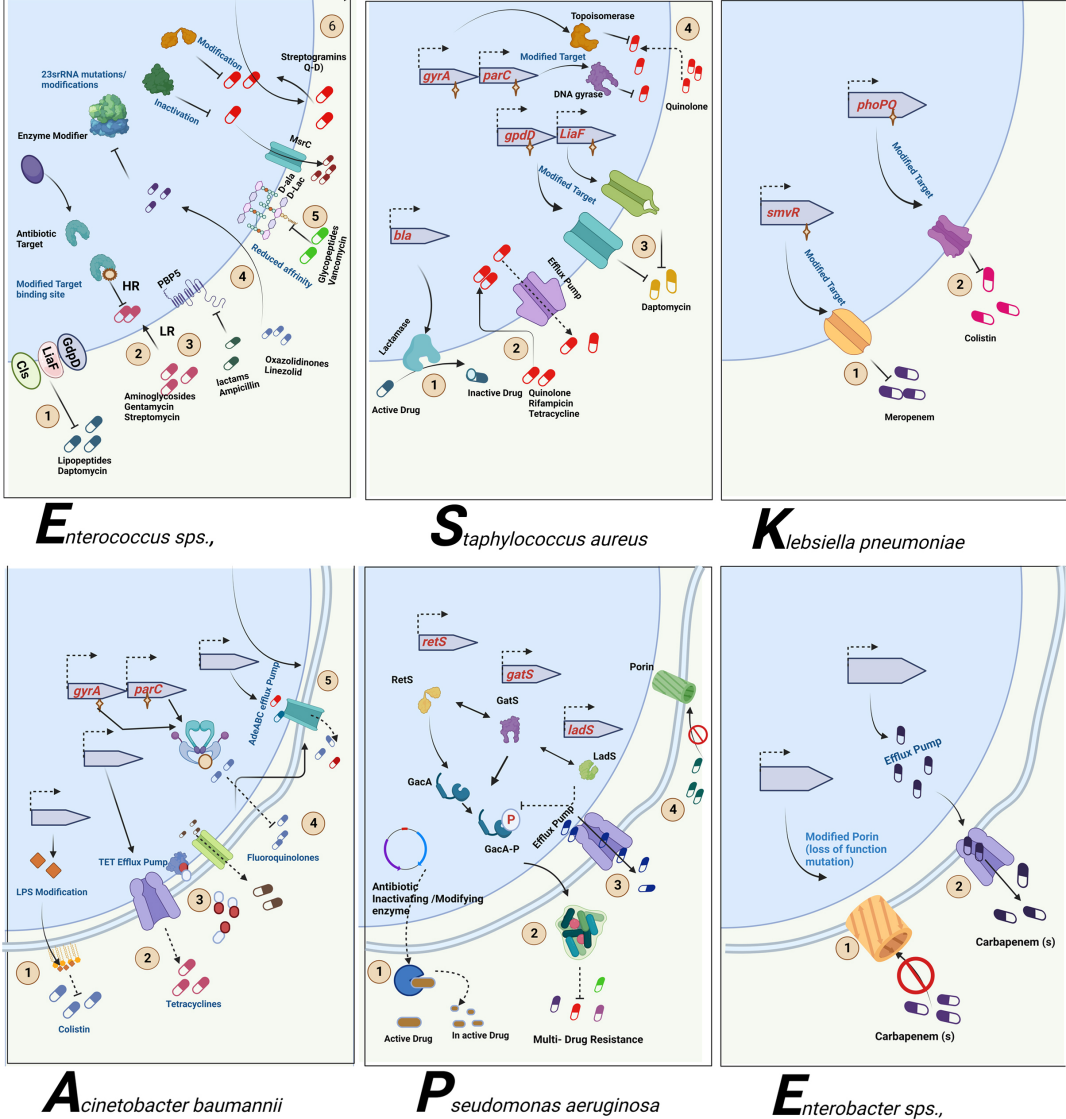
Antibiotic resistance mechanism of ESKAPE pathogens. ESKAPE pathogens have developed various antibiotic resistance mechanisms against the different classes of antibiotics ranging from aminoglycosides to carbapenems. The exact ways each of these pathogens develops and disseminates resistance through biofilms vary widely. However, the most common mechanisms include the overexpression of efflux pumps, modification of cell wall composition and permeability, modification of the target, inactivation of the antibiotics, and reduction in antibiotic penetration through biofilm formation. Created with BioRender.com.

As mentioned, the bacterial team has emerging mechanisms to overcome antibiotic stress. ESKAPE pathogens have the gene(s) employed for each class of antibiotics for the resistance mechanism. The primary class of antibiotics is β-lactams, aminoglycosides, chloramphenicol, glycopeptides, tetracyclines, oxazolidinones, macrolides, ansamycins, streptogramins, and lipopeptides. Each class of antibiotic has a specific mechanism of action against bacteria and, hence, an exact resistance mechanism. The typical resistance mechanisms are antibiotic-inactivating enzymes, overexpression of efflux pumps, modifications in the target site, and the acquisition of resistance genes through horizontal gene transfer (Bhukta et al., 2022) (**Figure 3**). **Table 2** elaborates on the specific set of genes essential for the resistance process of each antibiotic used.

**Table 2:** Summary of the antibiotics employed and the resistance mechanisms evolved by the ESKAPE pathogens

Class of antibiotic	Molecular target	Function of the targeted molecule	Organism	Resistance mechanism	Genes involved	References
β-Lactams	Penicillin-binding proteins (PBPs)	Synthesis of peptidoglycan	*Enterococcus* sp.	Alteration of PBPs	*pbp5*	(Beta-Lactam Antibiotics - an overview | ScienceDirect Topics; Miller et al., 2014; Maréchal et al., 2016)
				Production of β-lactamases	*pbp5*	
			*Staphylococcus aureus*	Alteration of PBPs	*pbp2*	(Hackbarth et al., 1995; Foster, 2017)
				Production of β-lactamases	*blaZ*	
			*Klebsiella pneumoniae*	Alteration of PBPs	*pbp2* and *pbp4*	(Lin et al., 2006; Sutaria et al., 2018)
				Production of extended-spectrum β-lactamases (ESBLs)	*shv-27* and *tem-116*	
			*Acinetobacter baumannii*	Alteration of PBPs	*ponA*, *mrcB*, *pbpA*, and *fts1*	(Cayô et al., 2011; Alkasaby and El Sayed Zaki, 2017; Abdi et al., 2020; Uppalapati et al., 2020)
				Alterations in outer membrane proteins (OMPs)	*ompA*, *carO*, and *oprD*	
				Production of extended-spectrum β-lactamases	*tem*, *shv*, and *ctx-m*	
				High activity of efflux pumps	*ade gene cluster*	
			*Pseudomonas aeruginosa*	Alteration of PBPs	*pbp2* and *pbp3*	(Pechère and Köhler, 1999; Giske et al., 2008; Poole, 2011)
				Alterations in permeability	*oprD*	
				Production of β-lactamases	*ampC* and *poxB*	
				High activity of efflux pumps	*mexAB-oprM*, *mexCD-oprJ*, and *mexXY-oprM*	
			*Enterobacter* sp.	Alteration of PBPs	*pbp3*	(Chen et al., 2017; Wu et al., 2018)
				Production of β-lactamases	*bla-shv12* and *bla-mir*	
Aminoglycosides	Ribosome	Bacterial protein synthesis	*Enterococcus* sp.	Aminoglycoside-modifying enzyme	*aph(2″)-Ib*, *aph(2″)-Ic*, and *aph(2″)-Id*	(Chow, 2000)
			*S. aureus*	Aminoglycoside-modifying enzymes (AMEs)	*aac(6′)-Ie +aph(2″*, ant(4’)Ia, *aph(3′)IIIa*, and *ant(6)-Ia*	(Rahimi, 2016)
			*K. pneumoniae*	Aminoglycoside-modifying enzymes (AMEs)	*aac(3)ii*, *aac (6′)-ib*, *ant (3″)-i*, and *ant (2″)-i*	(Liang et al., 2015)
			*A. baumannii*	Aminoglycoside-modifying enzymes (AMEs)	*aac(3)-i*, *aph(3′)-vi*, and *ant(3″)-i*	(Tahbaz et al., 2019)
			*P. aeruginosa*	Aminoglycoside-modifying enzymes (AMEs)	*aac(6′)-Ib*, *aphA1*, and *aadB*	(Teixeira et al., 2016)
			*Enterobacter* sp.	Ribosomal modification	*rmtE*	(Garneau-Tsodikova and Labby, 2016)
Chloramphenicol*	50S ribosomal subunit	Peptidyl transferase activity	*Enterococcus* sp.	Inactivation of chloramphenicol	*catA7*, *catA8*, and *catA9*	(Hasani et al., 2012)
			*S. aureus*	Inactivation of chloramphenicol	*cat genes*	(Genetics of Antimicrobial Resistance in Staphylococcus Aureus)
			*K. pneumoniae*	Inactivation of chloramphenicol	*catB3*, *catA1*, and *catA2*	(Mbelle et al., 2020)
			*A. baumannii*	Inactivation of chloramphenicol by the action of chloramphenicol acyltransferase	*ABUW_0982 of CHL gene cluster*	(Karalewitz and Millera, 2018)
			*P. aeruginosa*	Inactivation of chloramphenicol	*catB7*	(White et al., 1999)
			*Enterobacter* sp.	Efflux pumps	*AcrAB–TolC* and *eefABC*	(Davin-Regli and Pagès, 2015)
Glycopeptides	Peptidoglycan precursors	Synthesis of peptidoglycan, by preventing transglycosylation and transpeptidation	*Enterococcus* sp.	Change in the amino acid sequence of the precursor of peptidoglycan	*vanH*, *vanA*, and *vanZ*	(Miller et al., 2014)
			*S. aureus*	Modification of the target molecule	*pbp2*	(Foster, 2017; Yushchuk et al., 2020)
				Modification of the target	*vanA*	
			*K. pneumoniae*	–	*-*	–
			*A. baumannii*	–	*-*	–
			*P. aeruginosa*	Adhesin factor*//	*lecA*	(Pang et al., 2019)
			*Enterobacter* sp.	–	*-*	–
Tetracyclines*	30S ribosomal subunit	Bacterial protein synthesis	*Enterococcus* sp.	Efflux pumps	*tetM* and *tetL*	
			*S. aureus*	Efflux pumps	*tetA(K)* and *tetA(L)*	(Foster, 2017)
			*K. pneumoniae*	Efflux pumps	*tetA* and *tetB*	(Bokaeian et al., 2014)
			*A. baumannii*	Efflux pumps	*tetA* and *tetB*	(Maleki et al., 2014)
			*P. aeruginosa*	Efflux pumps	*tetR*, *lysR*, *marR*, and *araC*	(Issa et al., 2018)
			*Enterobacter* sp.	Efflux pumps	*AcrAB–TolC* and *eefABC*	(Davin-Regli and Pagès, 2015)
Oxazolidinones*	Ribosome	Bacterial protein synthesis	*Enterococcus* sp.	Alterations in oxazolidinone binding sites	G2576T mutation in the V domain of the 23S rRNA gene	(Chen et al., 2019)
			*S. aureus*	Alterations in oxazolidinone binding sites	U2500A and G2447U mutations in the 23S rRNA encoding gene	(Long and Vester, 2012)
			*K. pneumoniae*	PhoPQ‐governed lipid A remodeling	*mgrB* mutation	(Kidd et al., 2017)
			*A. baumannii*	Modification of target	Mutations in the 23S rRNA encoding gene	(Vrancianu et al., 2020a)
			*P. aeruginosa*	–	*-*	–
			*Enterobacter* sp.	Modification of target	G2576T mutations	(Deshpande et al., 2018)
				Mobile Genetic Elements	*optrA*	
Macrolides*	Ribosome	Bacterial protein synthesis	*Enterococcus* sp.	–	*–*	*–*
			Staphylococcu *S. aureus*	Modification of target	*erm*(B)	(Schmitz et al., 2000; Wolter et al., 2005; Taitt et al., 2014)
				Efflux pumps	*mef(*A), *msrA*, and *msrB*	
				Modification of binding site	Mutations in 23S rRNA and riboproteins L4 and L22	
			*K. pneumoniae*	–	–	–
			*A. baumannii*	Efflux pump	*adeRS*	(Vrancianu et al., 2020a)
			*P. aeruginosa*	Efflux pump	Mutation in *MexCD-OprJ*	(Pang et al., 2019)
			*Enterobacter* sp.	–	–	–
Ansamycins	RNA polymerase	Transcription	*Enterococcus* sp.	Modification of target	Substitution in *rpoB* gene	(Enne et al., 2004)
			*S. aureus*	Modification of target	Mutation in *rpoB* gene	(Wang C. et al., 2019)
			*K. pneumoniae*	Modification of target *//	*arr2*	(Tribuddharat and Fennewald, 1999; Arlet et al., 2001)
			*A. baumannii*	Modification of target	Mutation in *rpoB* gene	(Giannouli et al., 2012)
			*P. aeruginosa*	Modification of target	Mutation in *rpoB* gene	(Yee et al., 1996)
			*Enterobacter* sp.	Alteration of binding sites	Mutation in Rifampin resistance-determining region (RRDR)	(Weinstein and Zaman, 2019)
				Modification of target	Substitution in *rpoB* gene	
Streptogramins	23S rRNA of 50S ribosomal subunit	Bacterial protein synthesis	*Enterococcus* sp.	Alteration of binding sites	*erm*	(Hershberger et al., 2004)
			*S. aureus*	Alteration of binding sites	*ermA* and *ermC*	(Lina et al., 1999)
			*K. pneumoniae*	rRNA modification	*erm*	(Ogawara, 2019)
			*A. baumannii*	–	*-*	–
			*P. aeruginosa*	–	*-*	–
			*Enterobacter* sp.	Efflux pump	*Lsa*	(Poole, 2007)
Lipopeptides	Multiple targets	Multiple functions	*Enterococcus* sp.	Modification of cell envelope stress response	*LiaR*	(Arias et al., 2011; Tran et al., 2013; Reyes et al., 2015)
				Modification of membrane phospholipid mechanism	*Cls* and *GdpD*	
			*S. aureus*	Mutations in RNA polymerase	*rpoC* and *rpoB*	(Montera et al., 2008)
				Mutation in lysylphosphatid-ylglycerol synthetase	*mprF*	
				Mutation in histidine kinase	*yycG*	
			*K. pneumoniae*	–	*-*	–
			*A. baumannii*	Persister formation	Mutation in *ΔrelA*	(Monem et al., 2020)
			*P. aeruginosa*	–	*-*	–
			*Enterobacter* sp.	–	*-*	–

In some cases, resistance is caused when combinatorial therapy is employed. In fact, it is reported that certain combinations of antibiotics could induce resistance (Liu et al., 2020). Therefore, it is important to choose the right combination of antibiotics.

*Bacteriostatic activity.

"-" denotes insufficient information.

### β-Lactams

β-Lactams are one of the commonly administered drugs against bacterial infections. They target penicillin-binding proteins (PBPs) and carboxypeptidases involved in peptidoglycan synthesis. β-Lactams form a stable covalent complex with PBPs and stall the cell wall synthesis, leading to cell death. To overcome the survival pressures, bacteria have evolved to resist β-lactams by altering their proteins, producing β-lactam-degrading enzymes, and using excessive efflux pumps to efflux the antibiotics. In the case of *Enterococcus* sp., *pbp5* is responsible for altering PBPs and β-lactamase production (Miller et al., 2014; Maréchal et al., 2016). In *S. aureus*, *pbp2* gene is required for the protein alteration, whereas *blaZ* is responsible for β-lactamase (Hackbarth et al., 1995; Foster, 2017). *K. pneumoniae* utilizes *pbp2* and *pbp4* for altering PBPs and *shv-27* and *tem-116* for the production of ESBLs (Lin et al., 2006; Sutaria et al., 2018). *A. baumannii* alters PBPs using *ponA*, *mrcB*, *pbpA*, and *fts1*; *tem*, *shv*, and *ctx-m* for the production of ESBLs; *ompA*, *carO*, and *oprD* for the alteration of the outer membrane proteins; and *ade* gene cluster to accentuate the high efflux pump activity (Cayô et al., 2011; Alkasaby and El Sayed Zaki, 2017; Abdi et al., 2020; Uppalapati et al., 2020). *P. aeruginosa* and *Enterobacter* sp. rely on *pbp3* for the alteration of PBPs. *P. aeruginosa* employs *ampC* and *poxB* for the production of β-lactamases; permeability modification and high efflux pump activity are brought about by *oprD*, *mexAB-oprM*, *mexCD-oprJ*, and *mexXY-oprM* (Pechère and Köhler, 1999; Giske et al., 2008; Poole, 2011). *bla-shv12* and *bla-mir* of *Enterobacter* sp. are required to produce β-lactamases (Chen et al., 2017; Wu et al., 2018).

### Aminoglycosides

Aminoglycosides are broad-spectrum antibiotics that can be used against Gram-negative and Gram-positive pathogens. They are known to bind to ribosomes and affect the translation of proteins. Structurally, aminoglycosides (AGs) are 2-deoxystreptamine (2-DOS) attached with amino-modified sugars. Owing to their structure, bacteria have developed intrinsic resistance by lowering the AGs’ permeability through the modified bacterial cell wall. They also employ modifying enzymes: Aminoglycoside modifying enzymes (AME) and RNA methyltransferases. AMEs are the most common AG resistance operated by the ESKAPE group. These are family enzymes that inactivate an aminoglycoside at a specific position; hence, the gene responsible carries the modification site number. These enzymes are further divided into three classes based on the modification of substrates: AG *N*-acetyltransferases (AACs), AG *O*-nucleotidyltransferases (ANTs), and AG *O*-phosphotransferases (APHs). *aph(2″)-Ib*, *aph(2″)-Ic*, and *aph(2″)-Id* of *Enterococcus* sp. encode AG *O*-phosphotransferases majorly (Chow, 2000). In *S. aureus*, genes such as *aac(6′)-Ie +aph(2″)*, *ant(4′)Ia*, *aph(3′)IIIa*, and *ant(6)-Ia* are present and can target all three types of substrates (Rahimi, 2016). *K. pneumoniae* possesses genes *aac(3)ii*, *aac(6′)-ib*, *ant(3″)-i*, and *ant(2″)-i*, which focus on the AACs and ANTs (Liang et al., 2015). All three methyltransferases are present in *A. baumannii* encoded by *aac(3)-i*, *aph(3′)-vi*, and *ant(3″)-i* (Tahbaz et al., 2019). *P. aeruginosa* possesses genes *aac(6′)-Ib*, *aphA1*, and *aadB*, which are required for modifying AGs (Teixeira et al., 2016). In the case of *Enterobacter* sp., ribosomal modification is brought about by *rmtE* encoding ribosomal methyltransferase, which methylates the nucleotide G1405 at the N7 position and confers resistance to aminoglycosides (Garneau-Tsodikova and Labby, 2016).

### Chloramphenicol

Chloramphenicol is a broad-spectrum antibiotic that is extracted from *Streptomyces* sp. Depending on the concentration, chloramphenicol can be bacteriostatic and bactericidal. It binds to the 50S subunit of the ribosome, blocking the peptide bond formation and, thus, the protein synthesis. Enzyme inactivation is the standard mechanism of resistance to chloramphenicol, especially by chloramphenicol acetyltransferase (CAT). CAT inactivates chloramphenicol by modifying the 3-hydroxyl group through acetyl-*S*-CoA-dependent acetylation. Another means is through the overexpression of efflux pumps. In *Enterococcus* sp., three prominent genes, *catA7*, *catA8*, and *catA9*, encode CAT (Hasani et al., 2012). *S. aureus* cat genes are also prevalent in the MRSA strains (Udo et al., 2021). In *K. pneumoniae*, *catB3*, *catA1*, and *catA2* are expressed to inactivate chloramphenicol (Mbelle et al., 2020). In the case of *A. baumannii*, recent studies showed that mutations in *ABUW_0982* of the *CHL* gene cluster encoding permease contribute to the intrinsic resistance and thereby reduce the permeability of the chloramphenicol into the cell (Karalewitz and Millera, 2018). *catB7* gene in *P. aeruginosa* encodes CAT, leading to chloramphenicol resistance (White et al., 1999). In *Enterobacter* sp., efflux pumps are the primary cause of chloramphenicol resistance; mainly, *AcrAB–TolC* and *eefABC* encoded efflux pumps (Davin-Regli and Pagès, 2015).

### Glycopeptides

Glycopeptide antibiotics (GPAs) are specifically administered against Gram-positive pathogens as a last-resort treatment. GPAs are glycosylated cyclic or polycyclic peptides (non-ribosomal) found naturally and synthetically. GPAs prevent the crosslinking of the peptidoglycan layer by specifically binding to the peptidoglycan precursors (d-Ala-d-Ala dipeptide), leading to incomplete transpeptidation and transglycosylation in Gram-positive pathogens. The perturbation in the peptidoglycan synthesis leads to defective cell walls, thereby leading to cell death. Gram-negative pathogens intrinsically resist GPAs based on their cell wall composition. The resistance to GPAs is brought about by modifying the target, unlike the shared mechanism of altering the antibiotic. Among the GPAs, vancomycin resistance is most common and reported widely (Yushchuk et al., 2020). The dipeptide sequence, d-Ala-d-Ala, is replaced by d-Ala-d-Lac or d-Ala-d-Ser, leading to the reduced affinity of the GPAs to the precursors. The genes bring about such replacements—*vanH*, *vanA*, and *vanZ*—in the case of *Enterococcus* sp. (Miller et al., 2014). It is shown that vancomycin resistance to *S. aureus* is through horizontal gene transfer from *Enterococcus* sp., and genes *pbp2* and *vanA* are responsible for the modification of the target dipeptide (Foster, 2017; Yushchuk et al., 2020).

### Tetracyclines

Tetracyclines are broad-spectrum antibiotics used to treat Gram-positive and Gram-negative pathogens and protozoan parasites in some cases. They are natural products obtained from *Streptomyces* sp. Tetracyclines bind to 30S ribosomal subunit and interact with 16S rRNA, interfering with the peptide elongation process (Grossman, 2016). They are generally bacteriostatic, but in some cases, bactericidal activity is also reported (Tessier and Nicolau, 2013). Both extrinsic and intrinsic resistance mechanisms bring about resistance to tetracycline. The critical resistance processes are overexpression of efflux pumps, mutations in the tetracycline binding site, inactivation of tetracycline, and expression of tetracycline-specific ribosomal protection proteins. The tetracycline-specific efflux pumps belong to the major facilitator superfamily (MFS), which excludes tetracycline at a proton’s expense. In *Enterococcus* sp., *tetM* and *tetL* encode the genes responsible for tetracycline exclusion, while *tetK* and *tetL* are required for *S. aureus* (Foster, 2017). Tet(K) and Tet(L) are expressed in Gram-positive pathogens, which are antiporters of monovalent H^+^ having 14 transmembrane segments of α and β domains. In both *K. pneumoniae* and *A. baumannii*, *tetA* and *tetB* are present and encode the H^+^ antiporters having 12 transmembrane segments of α and β domains (Bokaeian et al., 2014). Tet(A) and Tet(B) are present mainly in Gram-negative pathogens (Maleki et al., 2014). *P. aeruginosa* possesses *tetR*, *lysR*, *marR*, and *araC* genes that encode the efflux pumps (Issa et al., 2018). In contrast, *acrAB–tolC* and *eefABC* also play a role in tetracycline efflux in *Enterobacter* sp. (Davin-Regli and Pagès, 2015). Tetracycline-specific ribosomal protection proteins (RPPs), having significant similarity to elongation factors EF-G and EF-Tu, bring about conformational change in the ternary complex and enable translation even in the presence of tetracycline (Dönhöfer et al., 2012). Inactivation of tetracycline is facilitated by *tet(X)* gene that encodes Tet(X) monooxygenase enzyme that inactivates tetracycline by the addition of hydroxyl group in C11 position of the tetracycline core (Aminov, 2013). Such RPPs and Tet(X) enzymes are found in ESKAPE pathogens, leading to tetracycline resistance.

### Oxazolidinones

Linezolid and tedizolid belong to oxazolidinones, synthetic drugs for treating Gram-positive pathogens resistant to other antibiotics. Gram-negative pathogens are also treated with these antibiotics in some cases. These bacteriostatic antibiotics inhibit protein synthesis by binding to the P site of the 50S ribosomal subunit (Bozdogan and Appelbaum, 2004). Development of resistance to oxazolidinones is rare, but reports show a common mechanism of resistance, unlike other antibiotics. Resistance is conferred by altering the oxazolidinone binding sites by mutations in 23S rRNA and acquiring mobile genetic elements (Brenciani et al., 2022). In *Enterococcus* sp., alterations in binding sites are through G2576T mutation in the V domain of the 23S rRNA gene (Chen et al., 2019), whereas in *S. aureus*, alterations in binding sites are through U2500A and G2447U mutations in the 23S rRNA encoding gene (Long and Vester, 2012). In *K. pneumoniae*, *mgrB* mutation leads to PhoPQ-mediated lipid A remodeling (Kidd et al., 2017). G2576T mutations that modify the target and *optrA* mobile genetic elements facilitate the resistance in *Enterobacter* sp. (Deshpande et al., 2018).

### Macrolides and streptogramins

Macrolides are a class of antibiotics that primarily target Gram-positive pathogens but also have been shown to possess broad-spectrum activity. Structurally, they have 14-, 15-, or 16-membered lactone rings having sugar moieties and other substitutions in the lactone ring. Macrolide antibiotics target protein synthesis by binding to large subunits, leading to cell growth arrest (Nakajima, 1999). The primary resistance mechanisms are modification of the target site, 23S rRNA, mediated by erm gene, overexpression of efflux pumps, and inactivation of the antibiotics through esterase and macrolide phosphotransferase enzymes. *erm* gene encodes Erm methyltransferase, which catalyzes the demethylation of the macrolide binding site leading to the reduced affinity brought about by stearic hindrance (Gaynor and Mankin, 2003). *S. aureus* to overcome macrolide pressure—*erm(B)*, *mef(*A), *msrA*, and *msrB* genes—to encode efflux pumps is present (Schmitz et al., 2000; Wolter et al., 2005; Taitt et al., 2014). *A. baumannii* overexpresses adeRS efflux pumps to reduce the accumulation of macrolides (Vrancianu et al., 2020b), whereas *P. aeruginosa* relies on the mutation in *MexCD-OprJ* efflux pumps (Pang et al., 2019). The other inactivating enzymes are not significantly reported in the clinical isolates.

A similar mechanism of action is followed by streptogramins, even though they are structurally diverse from macrolides. Streptogramins contain two subunits of distinct classes—type A and type B. They interfere with peptidyl transferase activity, inhibiting protein synthesis (Johnston et al., 2002). Individually, type A and type B are bacteriostatic, but they exhibit bactericidal activity when combined. Another commonality is the resistance mechanism against streptogramins—modification of target mediated by *erm* gene. Erm methyltransferase is present in *Enterococcus* sp. (Hershberger et al., 2004), *S. aureus* (Lina et al., 1999), and *K. pneumoniae* (Ogawara, 2019), leading to alteration of the target site and, thus, resistance. *Enterobacter* sp. uses *lsa* efflux pump to efflux out the streptogramins (Poole, 2007). Gram-negative pathogens are intrinsically resistant to streptogramins owing to the impermeability of their cell membrane.

### Ansamycins

Ansamycins are rigid antibiotics because they have an aromatic nucleus and a long aliphatic bridge with a handle shape. This unique configuration confers unique biological properties. They target RNA polymerase (RNAP) in bacteria, which is essential but also structurally diverse from humans. Ansamycins bind to RNAP near the catalytic site, leading to abortive transcription. Thus, modification of the target site is the primary resistance mechanism and mainly maps to the *ropB* mutation. These mutations are single amino acid substitutions pointing to a few deletions or mutations in the case of *Enterococcus* sp. (Enne et al., 2004), *S. aureus* (Wang C. et al., 2019), *A. baumannii* (Giannouli et al., 2012), *P. aeruginosa* (Yee et al., 1996), and *Enterobacter* sp. (Weinstein and Zaman, 2019). Other resistance mechanisms include *arr2* gene responsible for the inactivation of rifamycin through ribosylation (Tribuddharat and Fennewald, 1999; Arlet et al., 2001).

### Lipopeptides

Lipopeptides are a class of antimicrobials derived naturally from *Actinomyces*, *Bacillus*, and *Pseudomonas* sp. Structurally, they are made of hydrophilic peptides and attached to a fatty acyl chain, which is hydrophobic. They exist in linear and cyclic forms, with up to 25 amino acid chains (Patel et al., 2015). The most prominent lipopeptides like polymyxins, daptomycin, surfactin, iturin, and pseudofactin take the cyclic form. Even though the exact mechanism of action of lipopeptides is yet to be elucidated, studies have shown interactions with the bacterial cell membrane calcium (Ho et al., 2008), and phospholipid phosphatidylglycerol has been shown to play a role in the antimicrobial action. These interactions improve the access to lipopeptide antibiotics in the bacterial cell membrane, thereby interfering with the integrity of the cell membrane, leading to cell death. Lipopeptide antibiotics insert in the cell membrane form pore, extract the lipid in the membrane, and translocate the membrane. Thus, resistance mechanism developed by bacteria is focused on modifications in the cell membrane protein. Through physical repulsion, bacteria evade the incoming antibiotic. In *Enterococcus* sp., *liaR* gene modifies the cell envelope stress response, and *cls* genes that encode cardiolipin synthase decrease the surface charge of the membrane and modify the phospholipid composition (Arias et al., 2011; Tran et al., 2013; Reyes et al., 2015). The resistance mechanism against lipopeptides are studied extensively in *S. aureus*. It was found that the changes in surface charge and modification or overexpression of lipopolysaccharide layer forming septa are the major mechanisms of resistance. mprF mutation encoding lysyl phosphatidyl glycerol synthetase leads to gain-of-function and thereby increases synthesis of positive charged lipopolysaccharide. Mutation in histidine kinase yycG leads to increased glycan chain length (Montera et al., 2008).

## Alternate strategies: a way of escaping from ESKAPE pathogens

The bacterial team has seized the “ball of health” once again, despite recruiting new substitutes into the human team, which now cannot afford to increase the dosage of the recruited antibiotics due to the impending risk of toxicity. However, various alternate strategies are currently employed against the ESKAPE pathogens (**Table 3**; **Figure 1D**). Drug repurposing, where a drug used for another ailment, a previous-generation antibiotic currently in limited use or an orphan drug, is utilized as an antimicrobial agent, offers a new opportunity to invest in tuning up the strategies of the existing players. This is important, considering the significant time and money invested in identifying novel classes of antibiotics that are less prone to AMR (Silver, 2011). Modifying the functional groups helps build novel and effective antibiotics with the existing scaffold (Kamurai et al., 2020). Another quick-witted move along these lines is reinforcing combinatorial drugs with good chemistry in the team (Cheng et al., 2019). Adjuvants such as β-lactamase inhibitors prevent the degradation of the β-lactam antibiotics (Drawz and Bonomo, 2010; Ripoll et al., 2014), and efflux pump inhibitors inhibit the overexpressing efflux pumps, retaining the antibiotics to complete their course of action (Sharma et al., 2019; Verma et al., 2021), support the action of antibiotics by rendering a “double-attack defense”, and make it harder for the bacteria to shoot the target (González-Bello, 2017). Multiple strategies, such as monoclonal antibodies [which bind to the specific epitope of the bacterial cell targeting the conserved pathogenesis pathway and initiate immunological response leading to a second line of defense (Chames et al., 2009)], vaccines as a prophylactic tool to prevent the infection, and fecal microbiota transplant [one of the current trends where the stool from the healthy volunteer is transplanted into the patient helps in reversing the microbiome dysbiosis (Leshem et al., 2019)], are developed by tailoring specific drugs that target the rivals (Woodworth et al., 2019; Bekeredjian-Ding, 2020; Zurawski and McLendon, 2020). Consigning all-rounders like metal nanoparticles augments the team’s strength by targeting multiple mechanisms simultaneously (Borthagaray et al., 2018). There are multiple reports on the use of nanoparticles—metal, metal oxides, and polymeric—as a potential therapy to overcome the problem of resistance (Sharmin et al., 2021). Nanoparticles impart antibacterial activity at different levels: inhibit cell wall synthesis, inhibit biofilm, and target RNA and protein synthesis (Wang et al., 2017). The activity is achieved by increasing the reactive oxygen species that disintegrate the cell’s membrane potential (Slavin et al., 2017). These nanoparticles are also used as drug carriers for targeted action against pathogenic bacteria as against normal microbiota (Allahverdiyev et al., 2011). Silver, gold, zinc, copper, Cerium oxide, magnesium, chitosan, and cellulose-based nanoparticles are currently exploited as antimicrobials (Sánchez-López et al., 2020). Photo-antimicrobials are another interesting approach that combines the activity of dyes and light. Photo-antimicrobials absorb energy from the visible or infrared light and transfer it to molecular oxygen to generate reactive species—superoxide anions, singlet oxygen, and hydroxyl radicals—that can disrupt cells at multiple levels of proteins, lipids, and nucleic acids. Development of resistance is unlikely, as the target of action is not specific, and internalization of the drug is not mandatory in photodynamic therapy (Wainwright et al., 2017). To hold back the offending bacterial team, conjugation inhibitors and plasmid curing techniques are employed, which inhibit horizontal gene transfer and prevent the dissemination of the AMR genes into the bacterial community (Vrancianu et al., 2020b). Interestingly, taking inspiration from its opponents, the human team has been developing CRISPR-Cas-based systems to specifically compromise the antimicrobial resistant phenotype of the ESKAPE pathogens. Even though the guide-RNA based tool can be targeted against the virulent genes that contribute to antimicrobial resistance without affecting the natural microbiota, it comes with its own set of concerns including the possibility of off-target effects, reduced feasibility of the delivery system *in vivo*, and the involvement of the immune system (González de Aledo et al., 2021). Furthermore, various post-translational modifications (PTMs) of the ESKAPE pathogens could be targeted, considering their role in modulating the function of the proteins associated with bacterial virulence, motility, quorum sensing, and biofilm formation (Tiwari, 2019). Contrastingly, in the context of host–pathogen interactions, ESKAPE pathogens are reported to alter the PTMs of host proteins. Youssouf N et al. reported the ability of *S. aureus* to decrease the SUMOylation levels in the macrophages to enhance its chances of survival (Youssouf et al., 2021). One of the most elegant moves made by the human team is to recruit players with an excellent history of playing with the bacterial team. The involvement of bacteriophages in the game has proven to be a winning strategy because of its high specificity and efficiency (El Haddad et al., 2019). Phage therapy uses bacteriophages that infect pathogens as a treatment, which has been considered very potent in recent years. Precision medicine, i.e., phage preparations, can be performed for a specific set of clinical isolates that infect a patient. Phage cocktails and synergy with antibiotics are currently under consideration to prevent the development of resistance against phage therapy (Hatfull et al., 2022). In addition, lectin inhibition is considered promising, where naturally available lectins bind to the carbohydrates in the bacterial cell membrane. The interaction inhibits the invasion of the pathogen into the host and evokes the host’s immune response (Breitenbach Barroso Coelho et al., 2018). Along similar lines, essential oils have been shown to have antibacterial and anti-biofilm effects due to their ability to counter various virulence factors and quorum-sensing networks in ESKAPE pathogens. The ability to eradicate existing biofilms and their combinatorial effects on bacterial populations when employed with antimicrobials make them an attractive target (Panda et al., 2022). Iron chelation is also one of the promising approaches to overcoming antibiotic resistance. Iron is an important nutrient for pathogenic bacteria utilized for the essential growth and survival processes and in the host’s pathogenesis and invasion. Chelators (such as hydroxamates, catechols, and amino carboxylates) coordinate with Fe(III), reduce iron availability to the pathogens, and inhibit their growth (Vinuesa and McConnell, 2021). Several plant-based natural products are also exploited as antibacterial agents. Plants are a rich source of phytomolecules, which either alone or in combination impart antibacterial action against resistant pathogenic bacteria. They can act as efflux pump inhibitors, inhibit protein and nucleic acid synthesis, and disrupt cell membranes (Vaou et al., 2021). However, most of the strategies are at risk of inducing the onset of resistant phenotypes. However, immune boosters act as the energy drink for the human team and help build a strong defense, which complicates the process of scoring a goal by the bacterial team (**Figure 1**).

**Table 3:** Summary of the alternate strategies employed against the ESKAPE organisms and their limitations.

Alternate strategy	Function	Resistant organism(s)	Other limiting factor(s)	Reference(s)
β-Lactamase inhibitors	Prevent degradation of the β-lactam antibiotics	• *Enterococcus* sp. • *Klebsiella pneumoniae*	–	(Drawz and Bonomo, 2010; Ripoll et al., 2014)
Efflux pump inhibitors	Inhibit the efflux pumps, thereby localizing the antibiotics within the bacterial cell	–	• Difficulty in synthesizing the compounds • Solubility • Toxicity • Constraints in cell permeability • Drug compatibility	(Sharma et al., 2019)
Phage therapy	Employs bacteriophages to kill the pathogen	• *Enterococcus* sp. • *K. pneumoniae* • *Pseudomonas aeruginosa*	• Difficulty in tailoring the phage genome • Risk of inducing AMR • Hindrance of the immune response	(Oechslin, 2018; Principi et al., 2019)
Monoclonal antibodies	Bind to the specific epitope of the bacterial cell and instigate an immunological response	–	• Mode of action • Precise control of the characteristics like molecular size, shape, affinity, and valency	(Chames et al., 2009)
Vaccination	Prevents the corresponding bacterial infection	–	• Reversal of virulence, if live bacteria is employed as the vaccinating agent • Constantly mutating target*//	(Bacterial Vaccine - an overview | ScienceDirect Topics)
Fecal microbiota transplant	Aids in reversing dysbiosis by maintaining a healthy microbiome	–	• Difficulty in finding an ideal donor • Harmful microbial transfer to the donors • Colonization resistance	(Leshem et al., 2019)
Plasmid curing	Inhibits the conjugational transfer of the antibiotic-resistant plasmid	–	–	–
Conjugation inhibitors	Prevents horizontal gene transfer by inhibiting bacterial conjugation	–	–	–
Nanoparticles	• Target multiple mechanisms such as cell wall formation, biofilm formation, RNA, and protein synthesis. • Increase the production of reactive oxygen species (ROS) and disintegrates the membrane potential of the bacterial cell. • Trigger the host immune response systems	–	• Difficulty in ensuring surface stability and surface accessibility • Problems associated with optimizing the concentration	(Duval et al., 2019; Lee et al., 2019)
Antimicrobial peptides	Disrupt the membrane potential and alter the permeability of the bacterial cell wall	–	• Toxicity and stability	
Antimicrobial light therapy	Employs low-power lasers and photosensitive drugs to target the pathogens	–	–	–
Immune boosters	Stimulate the host immune system	–	–	–

“-” denotes insufficient information.

## Quorum sensing: the game changer

**Figure 4:**
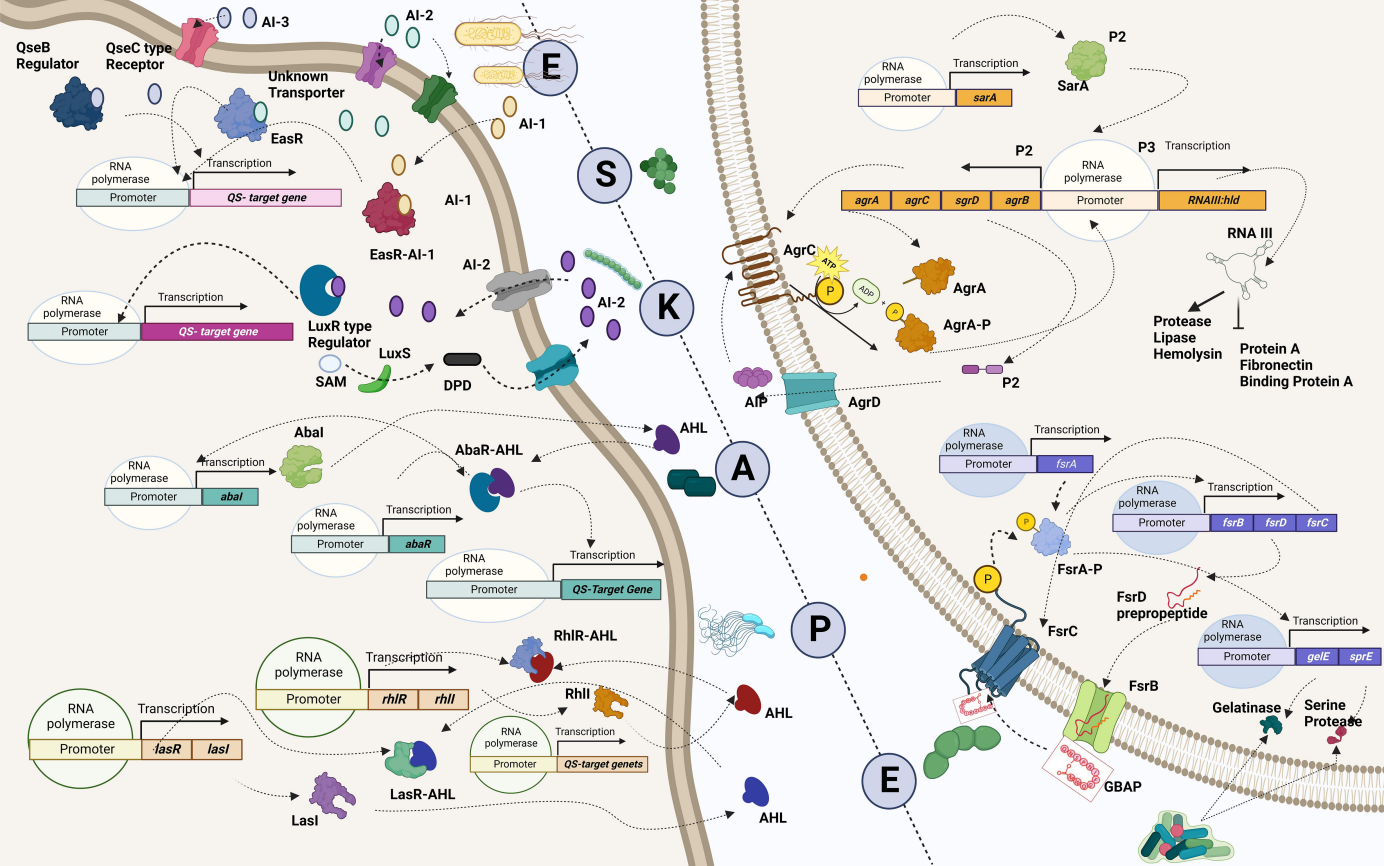
Quorum-sensing circuits of ESKAPE pathogens. All ESKAPE pathogens have been reported to have well-organized quorum-sensing circuits influencing their virulence and the ability to form biofilms. Four pathogens among the six, *Enterococcus* sp., *Staphylococcus aureus*, *Klebsiella pneumoniae*, and *Enterobacter* sp., involve LuxS system in altering antibiotic susceptibility and forming biofilms. More often than not, multiple quorum-sensing networks are involved in the biofilm formation process of these organisms. For instance, *Pseudomonas aeruginosa* is found to have a LasI–LasR system, RhII–RhIR system, and Quinolone and IQS systems in place to aid biofilm formation at various levels, including host tissue invasion and degradation. Similarly, the AbaI/AbaR system of *Acinetobacter baumannii* aids in its motility apart from contributing toward biofilm formation. Created with BioRender.com.

One major obstacle preventing the human team from winning is the development of resistance by the bacterial team to the opponent’s strategies. The bacterial team is well-founded in two fundamental needs to succeed in the game: it maintains a strong defense by forming a nearly impassable biofilm and devising new tactics in scoring a goal by developing virulence against the opponents (de Macedo et al., 2021). Building a team that is proficient in both requires good communication and co-operation. In the bacterial squad, this is ensured by quorum sensing, a mechanism that aids the bacterial players to coordinate among themselves to infect the humans (**Figure 4**) (Santhakumari and Ravi, 2019). While teamwork depicted by the bacterial players is crucial in escalating the game, the competency of individual species is also a significant driver. It is important to recall that the virulence factors that elevate the proficiency of the bacterial players are controlled by “quorum-sensing circuits” (**Table 4**). Understanding the various systems involved in quorum sensing is, therefore, crucial to upgrade the plans of the human team.

**Table 4:** Summary of the quorum-sensing systems employed by the ESKAPE organisms and the associated virulence factors.

Organism name	Quorum-sensing system	Major genes involved	Gene product	Function	Associated virulence factors	Molecule involved in quorum sensing	References
*Enterococcus* sp.	Fsr system	*fsrA*	FsrA	Regulates the expression of other genes of the *fsr* locus *fsrBCD*, *ef1097*, and protease coding genes *gelE-sprE*	• Degradation of host tissues • Regulation of autolysin *N*-acetylglucosaminidase (AtlA), which mediates eDNA release • Biofilm formation • Dissemination by mediating the cleavage of Ace protein	Gelatinase Biosynthesis Activating Pheromone (GBAP)	(Ali et al., 2017)
		*fsrB*	FsrB	Processes FsrD to produce GBAP			
		*fsrC*	FsrC	Transmembrane protein that senses the level of GBAP in the extracellular environment			
		*fsrD*	FsrD	Generates the propeptide FsrD, which later matures to form GBAP			
	LuxS system	*luxS*	LuxS	Cleaves *S*-ribosylhomocysteine into homocysteine and 4,5-dihydroxy-2,3-pentanedione (DPD), which is later cyclized to form AI-2	• Biofilm formation • TP generation, translation, cell wall/membrane biogenesis, and nucleotide transport and metabolism	Autoinducer-2 (AI-2)	(Ali et al., 2017)
	Cytolysin-mediated quorum sensing	*cylR1*	*CylR1*	Regulatory protein that binds to CylL_S_ at the membrane	• Ability to lyse the host cells	CylL_S_ and CylL_L_	(WO5_03151 - Cytolysin immunity protein CylI - Enterococcus faecalis EnGen0354 - WO5_03151 gene & protein; Ali et al., 2017)
		*cylR2*	*CylR2*	Represses the expression of cytolysin			
		*cylLL*	CylL_L_	Long subunit that autoinduces the expression of cytolysin			
		*cylLS*	*CylLS*	Short subunit that autoinduces the expression of cytolysin			
		*cylM*	*CylM*	Aids the post-translational modification of CylL_L_ and CylL_S_			
		*cylB*	*CylB*	Aids the processing and transport of CylL_L_ and CylL_S_			
		*cylA*	*CylA*	Activates CylL_L_ and CylL_S_ by cleaving 6 amino-acids			
		*cylI*	*CylI*	Acts as a cytolysin immunity protein			
*Staphylococcus aureus*	Agr (accessory gene regulator) system	*agrA*	AgrA	Regulates the expression of RNAII and RNAIII by binding to the respective promoters, P2 and P3. Also upregulates the expression of *psm*α and *psm*β operons (phenol-soluble modulins (PSMs))	• Expression of toxins, peptidases, hemolysin, and exoenzymes • Expression of adhesins • Protection from the immune system • Dissemination of the biofilm and colonization	Autoinducing peptide (AIP)	(Le and Otto, 2015)
		*agrB*	AgrB	A transmembrane endopeptidase that aids in the maturation and the export of AIP			
		*agrC*	AgrC	A transmembrane receptor protein that transduces the extracellular signal via AgrA			
		*agrD*	AgrD	Generates the precursor of autoinducing peptide (AIP)			
	LuxS system	*luxS*	LuxS	Aids the production of AI-2 and in the regulation of *cap* genes	• Capsule synthesis • Biofilm formation • Antibiotic susceptibility	Autoinducer-2 (AI-2)	(Zhao et al., 2010; Le and Otto, 2015)
*Klebsiella pneumoniae*	LuxS system	*luxS*	LuxS	Aids the production of AI-2	• Biofilm formation	Autoinducer-2 (AI-2)	(De Araujo et al., 2010; Chen L. et al., 2020)
*Acinetobacter baumannii*	AbaI/AbaR system	*abaI/abaR*	AbaI/AbaR	The autoinducing sensor protein AbaI generates AHL molecules, which can be bound by the AbaR receptors	• Biofilm formation • Motility	*N*-Acyl homoserine lactone (*AHL*)	(Saipriya et al., 2020)
*Pseudomonas aeruginosa*	LasI–LasR system	*lasI/R*	LasI and Las R	Upon activation, LasR–OdDHL and RhlR–BHL complexes further activate their expression by specifically binding to the promoter regions of *las/rhl* genes	• Biofilm formation • Expression of degradative enzymes like elastase, LasA protease, and alkaline protease • Production of exotoxins and hydrogen cyanide	*N*-Acyl homoserine lactones (HSL)-3oxo-C_12_	(Lee and Zhang, 2015)
	RhlI–RhlR system	*rhlI/II*	RhlI and RhlII		• Biofilm formation • Expression of degradative enzymes like rhamnolipids, pyocyanin, and elastase • Production of hydrogen cyanide	*N*-Acyl homoserine lactones (HSL)—C_4_	
	*Pseudomonas* Quinolone System	*pqsA*	*PqsA*	Anthranilate-coenzyme A ligase that aids the formation of anthraniloyl-coenzyme A by activating anthranilate, marking the first step in PQS biosynthesis	• Expression of pyocyanin and rhamnolipids	4-Quinolone	
		*pqsB*	*PqsB*	3-Oxoacyl-(acyl carrier protein) synthases that aid the formation of 2-heptyl-4-quinolone (HHQ), which acts as a precursor of PQS			
		*pqsC*	*PqsC*				
		*pqsD*	*PqsD*				
		*pqsE*	*PqsE*	Metallo-β-lactamase associated with PQS-mediated phenotypes			
		*pqsH*	*PqsH*	Flavin-dependent monooxygenase that hydroxylates HHQ			
	IQS system	*ambBCDE*	AmbBCDE	Non-ribosomal peptide synthase gene cluster involved in IQS synthesis that crosslinks external stress-related cues with various inter-cellular quorum-sensing networks	• Expression of degradative enzymes like elastase, pyocyanin, and rhamnolipids	2-(2-Hydroxyphenyl)-thiazole-4-carbaldehyde	
*Enterobacter* sp.	LuxS system	*luxS*	LuxS	QseA (quorum-sensing regulator A), and the LEE-encoded regulator, Ler	• Adhesion • Flagellin formation and motility	Autoinducer-2 (AI-2)	(Vendeville et al., 2005)

*Enterococcus* sp. is reported to have three quorum-sensing circuits: Fsr, LuxS, and cytolysis-mediated systems. The fsr system senses the presence of gelatinase biosynthesis-activating pheromone (GBAP), the matured form of the pro-peptide FsrD, through the transmembrane protein FsrC. FsrB aids the processing of FsrD. It also involves the FsrA protein, which regulates the expression of other genes of the *fsr* locus (*fsrBCD* and *ef1097*) and protease coding genes (*gelE-sprE*). Fsr system is implicated in degrading the host tissues, regulating the autolysin *N*-acetylglucosaminidase (AtlA) and, thereby, the release of eDNA, biofilm formation, and the cleavage of Ace protein and subsequent dissemination (Ali et al., 2017). The LuxS system, however, regulates cell wall biogenesis, nucleotide transport, and metabolism. It cleaves *S*-ribosyl homocysteine into homocysteine and 4,5-dihydroxy-2,3-pentanedione (DPD), which is later cyclized to form AI-2 (Ali et al., 2017). Finally, the ability to lyse the host cells is conferred by the cytolysin system (WO5_03151–Cytolysin immunity protein CylI–*E. faecalis* EnGen0354–WO5_03151 gene and protein; Ali et al., 2017). On the contrary, the Agr and LuxS systems are known to be employed by *S. aureus*. The accessory regulatory system (Agr in short) involves AgrD, which generates the autoinducing peptide (AIP) precursor, which acts as the quorum-sensing molecule. AgrB, a transmembrane endopeptidase, aids in the AIP’s maturation and export. At the same time, AgrC transduces the extracellular signal via AgrA, which is also implicated in the regulation of the expression of RNAII and RNAIII and the upregulation of *psm*α and *psm*β operons (phenol-soluble modulins (PSMs)). Signals associated with the Agr system influence the expression of toxins, peptidases, hemolysin, exoenzymes, and adhesins, in addition, to aiding in the protection from the immune system and the dissemination of the biofilm and colonization (Le and Otto, 2015). Furthermore, the LuxS system aids the production of AI-2 and regulates *cap* genes involved in capsule formation. It also affects biofilm formation and antibiotic susceptibility (Zhao et al., 2010; Le and Otto, 2015). A similar kind of LuxS networking is observed in *K. pneumoniae*, which aids in the production of AI-2 and enables biofilm formation (De Araujo et al., 2010; Chen L. et al., 2020). Biofilm-forming ability in *A. baumannii*, however, is reported to be influenced by the AbaI/AbaR system where the auto-inducing sensor protein, AbaI, generates *N*-acyl homoserine lactone (AHL) molecules, which can be bound by the AbaR receptors (Saipriya et al., 2020). Different quorum-sensing systems, including the LasI–LasR system, RhlI–RhlR system, *Pseudomonas* Quinolone System, and the IQS system, are reported in *P. aeruginosa*. Among these, the LasI–LasR system involves activated LasR–OdDHL and RhlR–BHL complexes, further activating their expression by specifically binding to the promoter regions of *las/rhl* genes, thereby regulating biofilm formation, production of exotoxins, and hydrogen cyanide. It is also reported to influence the expression of degradative enzymes like elastase, LasA protease, and alkaline protease. The RhII–RhIR system, however, is associated with the expression of degradative enzymes like rhamnolipids, pyocyanin, and elastase. It is also involved in the generation of hydrogen cyanide and biofilm formation. Alternatively, the *Pseudomonas* Quinolone System regulates the expression of pyocyanin and rhamnolipids. Finally, the IQS system is reported to be involved with a non-ribosomal peptide synthase gene cluster, which plays a role in IQS synthesis that crosslinks external stress-related cues with various inter-cellular quorum-sensing networks, thereby regulating the expression of degradative enzymes like elastase, pyocyanin, and rhamnolipids (Lee and Zhang, 2015). Finally, in *Enterobacter* sp., the LuxS system regulates adhesion, flagellin formation, and motility (Vendeville et al., 2005). In addition to facilitating bacterial virulence and biofilm formation, quorum-sensing molecules influence host–pathogen interactions. A recent study by Chakraborty et al. reported the hijacking role of 2-aminoacetophenone in altering the host autophagic and lipid biosynthesis mechanism in *P. aeruginosa*. Increased persistence of *P. aeruginosa* is attributed to the reduced expression of autophagy-mediating genes (Unc-51-like autophagy activating kinase 1 (*ULK1*) and *Beclin1*) and lipogenic gene [stearoyl-CoA desaturase 1 (*Scd1*)] (Chakraborty et al., 2023).

Insights on the quorum-sensing circuits have assisted the human team in advocating using quorum-sensing inhibitors (QSIs) as adjuvants to support the existing players—antibiotics and the immune cells (**Table 5**). Targeting one master player that supports and regulates other players is reported to be a successful strategy (Zhao et al., 2020). A gene knockout study involving LuxS/AI-2 deletion mutants observed reduced biofilm-forming ability in mutants compared to controls, thus proving the significant role played by the LuxS system in biofilm formation. This study, however, did not report any significant correlation between the proliferation ability of *Enterococcus* sp. and the absence of a functioning LuxS system (Yang et al., 2018). Another study involving a chemical inhibitor—siamycin I to block the *fsr* system of *Enterococcus* sp.—identified reduced growth, gelatinase activity, GBAP production, and biofilm-forming ability in the treated population in contrast to the control (Nakayama et al., 2007). Similarly, Balaban et al. reported reduced biofilm ability among the *S. aureus* population whose *agr* system was compromised (Balaban et al., 2007). Another study on *K. pneumoniae* reported decreased adherence and biofilm-forming ability of the chemically treated bacterial population as opposed to the controls with an effective C6-AHL system (Cadavid and Echeverri, 2019).

**Table 5:** Summary of the quorum-sensing inhibition methods employed against the ESKAPE organisms and their impact on pathogenicity.

Organism name	Method of disruption of quorum-sensing mechanism	Further details	Targeted quorum sensing system	Major parameters analyzed	Observations	References
*Enterococcus* sp.	Gene knockout	Long flanking homology (LFH) PCR was used to generate deletion mutants	LuxS/AI-2	Proliferation ability after deletion	No significant effect	(Yang et al., 2018)
				Biofilm-forming ability	Decreased	
	Chemical inhibitor	Siamycin I	*fsr* system	Growth of the microbe	Decreased	(Nakayama et al., 2007)
				Gelatinase activity	Decreased	
				GBAP production	Decreased	
				Biofilm-forming ability	Decreased	
*Staphylococcus aureus*	Gene knockout and chemical inhibitor	RIP	*agr* system	Biofilm-forming ability	Decreased	(Balaban et al., 2007)
*Klebsiella pneumoniae*	Chemical Inhibitor	2′-Hydroxycinnamic acid and 3-methyl-2(5H)-furanone	C6-AHL system	Biofilm-forming ability	Decreased	(Cadavid and Echeverri, 2019)
				Adherence	Decreased	
	Gene knockout	“Gene Gorging” method followed by allelic replacement with a kanamycin resistance-encoding gene (*Km*) was used to generate deletion mutants	LuxS/AI-2 system	Biofilm-forming ability	Decreased	(Chen L et al., 2020)
				Ability to synthesize lipopolysaccharide (*wzm)*	Decreased	
				Ability to synthesize polysaccharide (*pgaA*), which is involved in the synthesis of porin	Increased	
				Ability to synthesize type 3 fimbriae (*mrkA)*	No significant effect	
*Acinetobacter baumannii*	Chemical Inhibitor	Chloroquine, Levamisole, Propranolol, Erythromycin, and Azithromycin	Aba1/AbaR system	Biofilm-forming ability	Decreased	(Seleem et al., 2020)
				Twitching and swarming motilities	Decreased	
				Ability to produce proteolytic enzymes	Decreased	
				Resistance to oxidative stress	Decreased	
	Gene knockout	Cloned plasmid pMo130v was used to generate Δ*abaI* deletion mutants encompassing regions of the *A1S_0109* gene	Aba1/AbaR system	Biofilm-forming ability	Decreased	(Mayer et al., 2020)
				Surface associated motility	Decreased	
*Pseudomonas aeruginosa*	Chemical inhibitor	Catechin-7-xyloside (C7X), sappanol and butein	LasR system	Biofilm-forming ability	Decreased	(Zhong et al., 2020)
				Ability to generate pyocyanin	Decreased	
				Ability to generate rhamnolipids	Decreased	
				Ability to generate elastin	Decreased	
	Gene knockout and Chemical Inhibitor	Quercetin	LasI system	Biofilm-forming ability	Decreased	(Ouyang et al., 2020)
				Adhesion	Decreased	
				Swarming motility	Decreased	
			RhlI system	Biofilm-forming ability	No direct effect	
			LasI/RhlI system	Biofilm-forming ability	Decreased	
				Adhesion	Decreased	
				Swarming motility	Decreased	
*Enterobacter* sp.	–	–	–	–	–	–

"-" denotes insufficient information.

Furthermore, a knockout gene study on this bacterial species revealed the decreased ability to form biofilm and to synthesize lipopolysaccharide with almost no significant influence over the ability to synthesize type 3 fimbriae in deletion mutants (Chen L. et al., 2020). A similar observation of decreased ability to form biofilms and to produce proteolytic enzymes, resistance to oxidative stress, twitching, and swarming motilities occurred when *A. baumannii* was treated with a chemical inhibitor that influences the Aba1/AbaR system (Seleem et al., 2020). The decreasing trends in the biofilm-forming ability and the surface-associated motility were reported in the corresponding gene knockout models (Mayer et al., 2020). Along these lines, inhibition of the LasR system in *P. aeruginosa* decreased the ability to form biofilm and generate pyocyanin, rhamnolipids, and elastin (Zhong et al., 2020). Gene knockout analyses revealed the decreased biofilm-forming ability, adhesion, and swarming motility in LasI mutants (Ouyang et al., 2020). It can be concluded that quorum sensing is quintessential in regulating virulence factors. Therefore, targeting the quorum-sensing networks can help counter the virulent traits of the ESKAPE pathogens.

QSIs have proven instrumental in cheating bacterial players by obstructing communication. Interfering with communication has aided in reducing the team’s strength by compromising its ability to form biofilms and to express the associated virulence factors (Munir et al., 2020). This, in turn, has boosted the chances of antibiotics and the immune cells in tackling the individual bacterial players (Brackman et al., 2011) (**Figure 1F**).

It is important to note that most of the substitutes in the human team resorted to conferring selective pressure against the bacterial squad, which is not the case with QSIs (Rasmussen and Givskov, 2006). A competition study by Gerdt et al. showed that the inadequacy of quorum-sensing signals by QSI-sensitive bacteria and their cheating mechanisms against the rare QSI-resistant bacteria would inherently reduce the spread of resistance against QSIs targeting QS receptor function (Gerdt and Blackwell, 2014). It is therefore perceived to be a safer move by the human team, as it does not come with an inherent risk of development of resistance by the bacterial players (Zhou et al., 2020).

## Conclusion

The profound strategies employed by both teams make it equally hard for the opponent to win this never-ending “game of health”. However, understanding the opponent’s action plans would benefit the human team in devising holistic game plans. Employing quorum-sensing inhibitors along with specific antibiotics could prove to be an excellent combinatorial therapy in improving the chances of the human team winning by aiding the immune cells. However, the question of the efficacy of such combinations in treating well-established infections is yet to be addressed. Understanding the quorum-sensing signals might help us unravel the relationship between pathogens and normal microbiota of the host in disease progression in addition to answering the questions: i) do quorum-sensing signals of the pathogens aid in building a confluent microenvironment within the host? ii) Do the pathogens’ quorum-sensing signals influence the host’s natural microbiota? iii) Quorum-sensing signals ensure communication among a wide range of bacterial and fungal species. How can the pathogens be targeted with high specificity? Does the non-specific nature of QSIs disrupt the communication of normal microbiota, thereby exacerbating the condition? Recent studies report the development of resistance against quorum-sensing inhibitors. Therefore, the human team should constantly be vigilant to detect traces of resistance or “escaping” mechanisms that the bacterial players might develop.

## Author contributions

APS and PN conceived the idea. PV, SV, and HD designed and drafted the manuscript. APS, AS, and KS provided the illustrations for the figures. APS and PN proofread the manuscript and suggested critical changes. All authors contributed to the article and approved the submitted version.
